# Restoration of the GTPase activity and cellular interactions of Gα_o_ mutants by Zn^2+^ in *GNAO1* encephalopathy models

**DOI:** 10.1126/sciadv.abn9350

**Published:** 2022-10-07

**Authors:** Yonika A. Larasati, Mikhail Savitsky, Alexey Koval, Gonzalo P. Solis, Jana Valnohova, Vladimir L. Katanaev

**Affiliations:** ^1^ Translational Research Centre in Oncohaematology, Department of Cell Physiology and Metabolism, Faculty of Medicine, University of Geneva, CH-1211 Geneva, Switzerland.; ^2^ Institute of Life Sciences and Biomedicine, Far Eastern Federal University, 690090 Vladivostok, Russia.

## Abstract

De novo point mutations in *GNAO1*, gene encoding the major neuronal G protein Gα_o_, have recently emerged in patients with pediatric encephalopathy having motor, developmental, and epileptic dysfunctions. Half of clinical cases affect codons Gly^203^, Arg^209^, or Glu^246^; we show that these mutations accelerate GTP uptake and inactivate GTP hydrolysis through displacement Gln^205^ critical for GTP hydrolysis, resulting in constitutive GTP binding by Gα_o_. However, the mutants fail to adopt the activated conformation and display aberrant interactions with signaling partners. Through high-throughput screening of approved drugs, we identify zinc pyrithione and Zn^2+^ as agents restoring active conformation, GTPase activity, and cellular interactions of the encephalopathy mutants, with negligible effects on wild-type Gα_o_. We describe a *Drosophila* model of *GNAO1* encephalopathy where dietary zinc restores the motor function and longevity of the mutant flies. Zinc supplements are approved for diverse human neurological conditions. Our work provides insights into the molecular etiology of *GNAO1* encephalopathy and defines a potential therapy for the patients.

## Teaser

Encephalopathy mutations destabilize Gα_o_ nucleotide binding, structure, and signaling, features corrected by dietary zinc.

## INTRODUCTION

Heterotrimeric G proteins are the immediate cytoplasmic signaling transducers of G protein–coupled receptors (GPCRs), the largest receptor class in animals and the major target of modern drugs (*1*). Composed of the Gα, β, and γ subunits, they interact with receptors when the Gα is loaded with GDP (guanosine diphosphate) to undergo the activated GPCR-induced GDP-to-GTP (guanosine triphosphate) exchange and dissociation into the Gα-GTP and Gβγ components, both competent to transmit the signal further downstream (*2*). With time, intrinsic guanosine triphosphatase (GTPase) activity of the Gα subunits leads to GTP hydrolysis; the activity further sped up by the dedicated RGS (regulator of G protein signaling) proteins (*3*). The resultant Gα-GDP can reload with GTP (*4*) or complex back with Gβγ, thus closing the G protein activation-deactivation loop (*2*).

Of the 16 human Gα subunits, Gα_o_ is the major neuronal representative, transmitting the signals from numerous GPCRs in developing and adult brain. In 2013, the first cases were reported on patients with pediatric encephalopathy harboring de novo mutations in *GNAO1*, the gene encoding Gα_o_ (*5*). This discovery was followed by an avalanche of clinical analyses that cumulatively led to the recognition of *GNAO1* encephalopathy as a spectrum of neurodevelopmental disorders manifesting as motor dysfunction, epileptic seizures, and developmental delay first appearing mostly in infancy (*6*–*8*). Although Gα_o_, in addition to neurons, is also strongly expressed in glial cells (www.proteinatlas.org), disease modeling in mice (*9*) and brain organoids (*10*) demonstrated strong effects of *GNAO1* mutations on neuronal rather than glial differentiation, highlighting neurons as the main target of Gα_o_ malfunctioning in the disease. Gα_o_ couples to many neuronal GPCRs, such as D2 dopamine, μ-opioid, M2 muscarinic, α2-adrenergic, and more. As these GPCRs belong to the inhibitory receptors, Gα_o_ misfunctioning induced by mutations may be expected to disbalance the equilibrium formed by the stimulatory and inhibitory GPCR signaling and thus contribute to the disease manifestations (*11*).

**Fig. 1.**
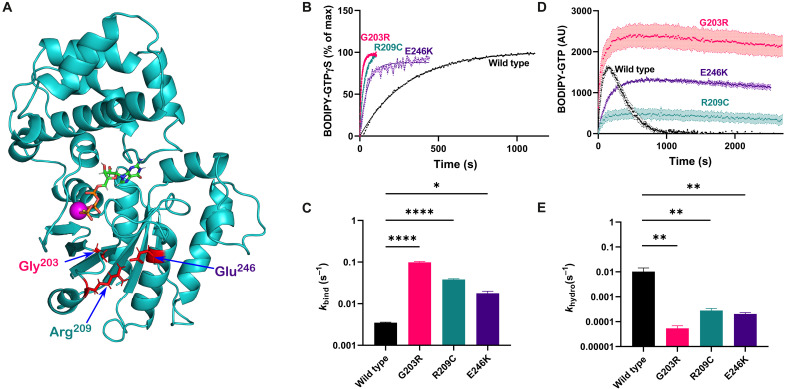
Mutations in positions 203, 209, and 246 result in constitutive GTP loading of Gα_o_. (**A**) Mutated amino acid residues (in red) in the overall structure of Gα_o_. The residues are either located in the switch II region of Gα_o_ (Gly^203^) or represent the molecular latch fastening switch II and the α3 helix (Arg^209^, Glu^246^), performing key functions in uptake and hydrolysis of GTP (shown as a stick structure with standardly colored atoms, complexed with magnesium in magenta). (**B** and **C**) Representative curves (B) and quantification of the binding rate constant (*k*_bind_) (C) of BODIPY-labeled GTPγS binding to Gα_o_, wild type (WT) or mutated. All the mutants demonstrate strongly elevated rates, with G203R being the fastest. (**D** and **E**) Representative curves (D) and quantification of the hydrolysis rate constant (*k*_hydr_) (E) characterizing the course of BODIPY-labeled GTP binding and hydrolysis by Gα_o_ or its mutants by monitoring the formation and decay of the GTP-bound fraction of Gα_o_. Note the difference between (B) where the data are adjusted to the plateau to highlight the differences in the binding rates and (D) where the data are shown in raw fluorescence units, as needed for the proper *k*_hydr_ calculation. Note the log scale in the *y* axes in (C) and (E). Data in (B) to (E) are shown as means of at least three biological replicates ± SEM. *P < 0.05; **P < 0.01, and ****P < 0.0005 by one-way analysis of variance (ANOVA) followed by Dunnett’s multiple comparisons test.

As of today, about 200 patients worldwide have been identified to harbor a mutation in *GNAO1* (https://gnao1.org/). With the advances in genetic analysis application in clinical practice, the reported *GNAO1* encephalopathy cases will continue to multiply. While most of the mutations being single–amino acid substitutions spread over the coding sequence of the gene, the codons Gly^203^, Arg^209^, and Glu^246^ emerge as the disease mutation hotspots, together taking the share of 45 to 68% in recent surveys (*6*–*8*). Of these amino acid residues, Gly^203^ is located near the GTP-binding pocket of Gα_o_, while Arg^209^ and Glu^246^ form a salt bridge, playing an important role in the adoption of the activated G protein conformation (Fig. 1A) (*12*).

No curative therapy exists for patients with *GNAO1* encephalopathy. Symptomatic treatments for motor dysfunctions include, e.g., benzodiazepines and deep-brain stimulation, while, for epilepsy, treatments include antiepileptic drugs such as carbamazepine, levetiracetam, or valproic acid, but at best with partial effects (*8*, *13*, *14*). Development of eventual therapies, in turn, is delayed by the lack of understanding of the molecular etiology of the disease. Here, we probe the biochemical deficiency at the molecular core of *GNAO1* encephalopathy, identify a drug correcting this deficiency, validate it in neuronal cells, and lastly show that the dietary supplementation of the drug rescues defects in a *Drosophila* model of the disease, identifying the potential therapeutic avenue to treat human patients.

## RESULTS

### Mutations in Gly^203^, Arg^209^, and Glu^246^ result in constitutive GTP binding by Gα_o_ in vitro

To seek understanding of the molecular etiology of *GNAO1* encephalopathy, we focused on the most frequent pathologic Gα_o_ mutants and first probed their basic biochemical properties: GTP uptake and hydrolysis, in comparison to the wild-type protein. The GTP uptake analysis has so far been performed for three *GNAO1* encephalopathy mutants: Q52P and Q52R displayed complete loss of the GTP uptake (*14*), while R209H was reported to display a faster GTP uptake (*15*). We applied the nonhydrolyzable fluorescent BODIPY-GTPγS (guanosine 5′-[γ-thio]triphosphate) (*4*, *16*–*18*) to monitor the GTP uptake properties of the wild-type protein and three Gα_o_ mutant variants: G203R, R209C, and E246K. This analysis reveals that the three mutants are much faster in uptaking GTP than the wild type (Fig. 1B). The calculated binding rate constant, *k*_bind_, increases 5-fold by the E246K mutation, 11-fold by the R209C mutation, and 28-fold by the G203R mutation over that of the wild-type Gα_o_ (Fig. 1C).

The faster GTP uptake will lead to higher GTP residence of the G protein if its GTP hydrolysis rate is not proportionally increased; an accompanying decreased GTP hydrolysis will further aggravate the GTP residence of the G protein. We thus next applied a hydrolyzable fluorescent GTP analog, BODIPY-GTP, whose interaction with an active G protein is seen as a transient rise in fluorescence (indicative of the nucleotide binding) followed by a decay in fluorescence (indicative of GTP hydrolysis due to the lower quantum yield the resultant fluorophore-GDP on the protein) (*4*, *17*, *18*). We see that all three pathologic Gα_o_ mutants reveal essentially abolished GTP hydrolysis as compared to the wild type (Fig. 1D). Calculation of the hydrolysis rate constant, *k*_hydr_, confirms this assessment: *k*_hydr_ of R209C is reduced ca. 50-fold, of E246K ca. 100-fold, and of G203R ca. 300-fold as compared to the wild-type Gα_o_ (Fig. 1E).

Thus, mutations in the three most frequently affected *GNAO1* encephalopathy amino acid residues lead to a strong increase in the rate of GTP uptake accompanied by a gigantic drop in the rate of GTP hydrolysis. We thus must conclude that, biochemically, mutations in Gly^203^, Arg^209^, and Glu^246^ of Gα_o_ lead to the constitutive GTP-binding state of the G protein, the molecular feature possibly at the basis of the etiology of the disease caused by these mutations.

### Defective cellular interactions of the *GNAO1* encephalopathy mutants

**Fig. 2.**
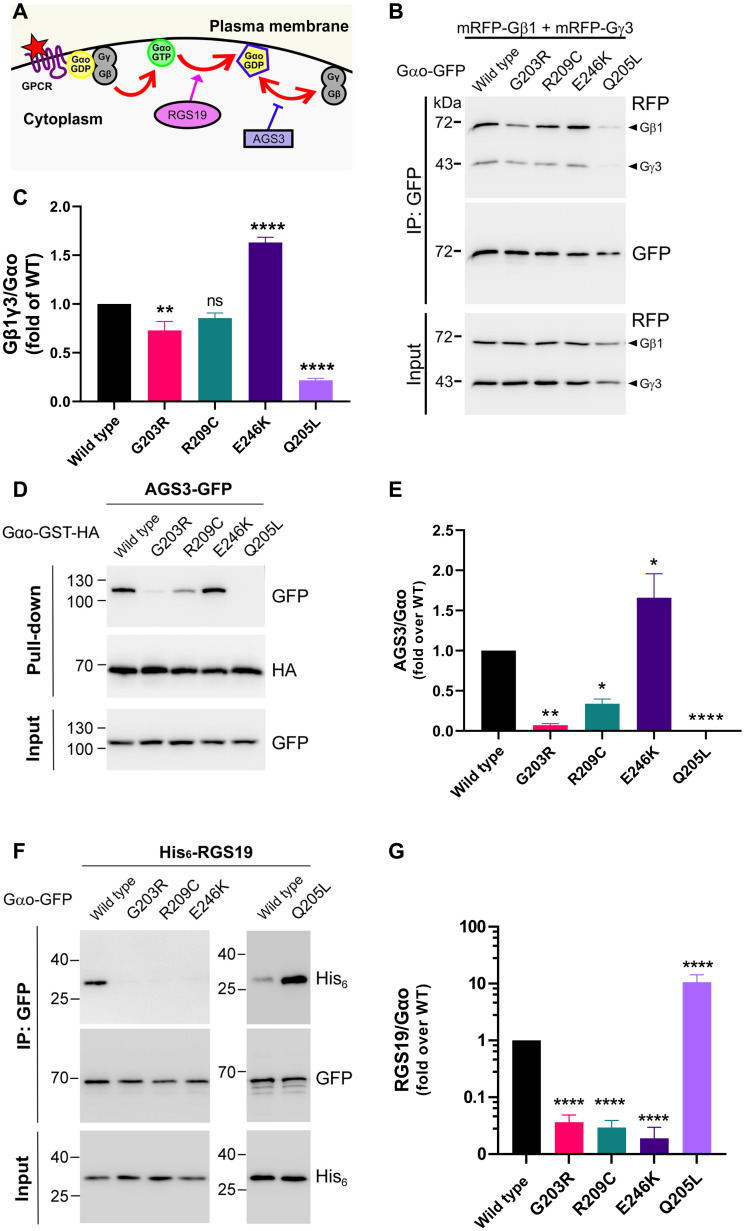
Aberrant cellular interactions of pathologic Gα_o_ mutants. (**A**) Analysis of plasma membrane/cytoplasmic partners of Gα_o_: Gβγ forming, with GDP-loaded Gα_o_, the heterotrimeric complex, competent to interact with a GPCR and respond to its activation by the GDP-GTP exchange and heterotrimer dissociation, RGS19 that speeds up GTP hydrolysis on Gα_o_, and AGS3 that competes with Gβγ for the GDP-loaded Gα_o_. Illustration modified from (*16*). (**B** and **C**) N2a cells were cotransfected with mRFP-Gβ1, mRFP-Gγ3, and Gα_o_-GFP (internally tagged) WT, G203R, R209C, E246K, or the activated Q205L mutant as control. Immunoprecipitation (IP) of Gα_o_ was done using a nanobody against GFP, and the coprecipitation of Gβ1γ3 was analyzed by SDS–polyacrylamide gel electrophoresis (SDS-PAGE) and Western blot (B). Antibody (Ab) against GFP was used for detection of Gα_o_ and against mRFP for Gβ1γ3 (arrowheads). Quantification of the coimmunoprecipitation (co-IP) of Gβ1γ3 by the different Gα_o_ constructs (C). (**D** and **E**) After cotransfection with AGS3-GFP and Gα_o_-GST-HA (C-terminally tagged) variants, pull-down of Gα_o_ was done using glutathione beads and samples were analyzed by SDS-PAGE and Western blot (D). Ab against hemagglutinin (HA) was used for detection of Gα_o_ and against GFP for AGS3. Quantification of the coprecipitation of AGS3-GFP by Gα_o_-GST-HA WT or mutants (E). (**F** and **G**) After cotransfection with His_6_-RGS19 and Gα_o_-GFP (internally tagged) variants, IP of Gα_o_ was done using a nanobody against GFP, and the coprecipitation of RGS19 was analyzed by SDS-PAGE and Western blot. Ab against GFP was used for detection of Gα_o_ and against His_6_ for RGS19 (F). Quantification of the co-IP of RGS19 by the different Gα_o_ constructs (G). Data in (C), (E), and (G) are means of ≥4 biological replicates ± SEM. Statistical significance: One-way ANOVA followed by Dunnett’s multiple comparisons test; *P < 0.05, **P < 0.01, and ****P < 0.0001.

The distorted proportion of the GTP/GDP-state residence of the *GNAO1* encephalopathy mutants inferred from the biochemical experiments must have consequences at the cellular level. We thus moved to express the mutants in mouse neuroblastoma Neuro-2a (N2a) cells, frequently used to study the Gα_o_ function (*16*). In this, as other cell lines, wild-type Gα_o_ shows dual localization at the plasma membrane and the Golgi apparatus, as previously reported by us (*16*, *19*). The G203R, R209C, and E246K mutants show a similar dual localization (fig. S1, A to D), indicative of the normal posttranslational modifications of the mutants (*20*). Quantification of fluorescence intensities at the two compartments confirmed the near-equal distribution of wild-type Gα_o_ and the three pathologic mutants (fig. S1, E and F). We next tested the interactions of the three pathologic Gα_o_ mutants, along with the wild-type and the classical constitutively activated, disease-unrelated point mutant Gα_o_[Q205L], with a set of intracellular binding partners of Gα_o_. The following interaction/signaling partners were selected: RGS19 that preferentially binds the GTP-loaded form (*4*), Gβγ that preferentially interacts with the GDP-loaded form of Gα_o_ (*21*), and AGS3 that also preferentially binds the GDP-loaded form (Fig. 2A) (*17*). We additionally tested the Golgi partners of Gα_o_: adenosine diphosphate ribosylation factor 1 (Arf1), Rab3a, and KDEL receptor (KDELR) (fig. S2A) (*16*). This broad assessment reveals a complicated nature of distortions of cellular interactions of the three pathologic Gα_o_ mutants. First, we found that interactions with the Golgi partners were near normal for the pathologic mutations (fig. S2, B to G). Second, we found that interactions with Gβγ were differentially affected by the three pathologic mutations: decreased for G203R, insignificantly affected for R209C, and unexpectedly increased for E246K (Fig. 2, B and C). Third, another GDP-preferring partner of Gα_o_, AGS3, revealed the same tendency with even more pronounced effects: decreased binding to Gα_o_[G203R] and Gα_o_[G209C] and enhanced to Gα_o_[E246K] (Fig. 2, D and E).

However, the most notable and unexpected was the near-complete loss of the pathologic mutants’ interaction with RGS19, opposite to that seen for the constitutively active Q205L (Fig. 2, F and G). In these pull-downs, we used an internal green fluorescent protein (GFP) fusion [that does not impede the proper localization nor protein-protein interactions (*14*)] of the Gα_o_ variants and a nanobody against GFP. To confirm that this placement of the GFP fusion has no decisive role in the markedly reduced interaction of the pathologic mutants with RGS19, we repeated the pull-downs with the C-terminally GFP-tagged Gα_o_ variants, again revealing that the *GNAO1* encephalopathy mutants lose the interactions with RGS19 (fig. S3, A and B).

Our findings may suggest that the pathologic Gα_o_ mutants fail to adopt the proper conformation upon nucleotide binding (see Discussion). Gα_i1_ mutated in the amino acids equivalent to Gα_o_’s Arg^209^ and Glu^246^, as well as at Gly^204^ neighboring Gly^203^, has similarly been proposed to fail to adopt the activated conformation and to dissociate from Gβγ upon GTP binding (*12*). Failure to adopt the properly activated conformation upon GTP binding by a Gα protein has also been associated with the reduced ability to hold the GTP nucleotide after binding (*12*). To assess this feature, we prebound Gα_o_ (wild type, G203R, R209C, and E246K or the constitutively active Q205L mutant) with BODIPY-GTPγS and then added excess of GDP, following (*4*, *12*, *17*). While wild-type and Q205L Gα_o_ retain a substantial portion of the prebound BODIPY-GTPγS (fig. S4, A and B), the three pathologic mutants lose most of it (fig. S4, C to F). Furthermore, the rate of dissociation of BODIPY-GTPγS from the three pathologic mutants is strongly increased as compared with the wild type or Gα_o_[Q205L] (fig. S4, A to E and G). Importantly, this rate of BODIPY-GTPγS dissociation significantly exceeds the rate of BODIPY-GTPγS uptake by the three pathologic mutants, the feature not seen for the wild-type and Q205L Gα_o_ (fig. S4G). These observations cumulatively argue that the three pathologic mutant forms of Gα_o_ do not acquire the proper conformation upon GTP binding and potentially upon nucleotide binding in general.

### Defective cellular GPCR signaling by the *GNAO1* encephalopathy mutants

Aberrant cellular interactions of the G203R, R209C, and E246K mutants of Gα_o_ described in the previous section suggest that some signaling routes mediated by the G protein could become aberrant in *GNAO1* encephalopathies caused by these mutations to different extents: potentially more at the plasma membrane and potentially less in the Golgi. We thus next aimed at investigation of how efficiently different neuronal GPCRs known to couple to Gα_o_ could activate signaling through these pathologic mutant variants.

**Fig. 3.**
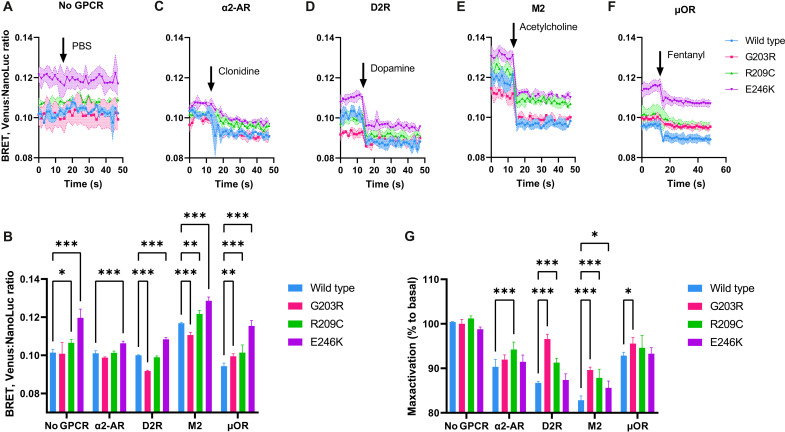
Aberrant signaling from neuronal GPCRs by pathologic Gα_o_ mutants. (**A**) BRET ratio between Gα_o_ (WT or mutant) tagged with nanoluciferase (NanoLuc) and Gβγ (Gβ3 and Gγ9 with the Venus tag) demonstrates different extent of association with Gβγ. Without cotransfection with a GPCR and upon a mock stimulation with phosphate-buffered saline (PBS), no change in the BRET signal is observed. (**B**) Quantification of the basal, nonstimulated levels of BRET (average of first 10 readings before agonist injection) for different Gα_o_ variants in different receptor cotransfection settings. (**C** to **F**) When the proper neuronal GPCR is cotransfected, the addition of the respective agonist (arrow) induces a robust decrease in the BRET signal, indicative of the dissociation of the Gα_o_-βγ heterotrimer, reduced to a different extent for different GPCRs. The agonist concentrations were 10 μM clonidine for the α2-adrenergic receptor (α2-AR) (C), 10 μM dopamine for the D2 dopamine receptor (D2R) (D), 10 μM acetylcholine for the M2 muscarinic receptor (M2) (E), and 75 nM fentanyl for the μ-opioid receptor (μOR) (F). (**G**) Quantification of the plateau levels of the BRET decrease upon GPCR stimulation (ratio of 10 readings at the end of stimulation to 10 readings before stimulation). Data on all graphs are presented as a means from *N* = 4 independent repeats; SEMs are represented by colored zone in (A) and (C) to (F) and by bars in (B) and (G). Statistical analysis is done by two-way ANOVA with Sidak corrections for multiple comparisons; *P < 0.05, **P < 0.01, and ***P < 0.001.

To this end, we used a bioluminescence resonance energy transfer (BRET) system in human embryonic kidney (HEK) 293 cells. Cell transfection with Gα_o_ (wild type or mutant) with the nanoluciferase tag, Gβ3, and Gγ9 with the Venus tag (*22*) results in a measurable BRET signal, indicative of the efficiency of the Gα_o_-βγ complex formation. Agreeing with the pull-down data presented above (Fig. 2, B and C), Gα_o_[E246K] demonstrates significantly higher BRET signal and, thus, Gα_o_-βγ complex formation than the wild type or the other two mutants (Fig. 3, A and B).

We next cotransfected the cells with neuronal GPCRs (D2 dopamine, μ-opioid, M2 muscarinic, or α2-adrenergic). Cell activation with respective GPCR agonists (dopamine, fentanyl, clonidine, or acetylcholine) results in a rapid decrease in the BRET, indicative of the GPCR-induced Gα_o_-βγ heterotrimer dissociation as the first step in activation of the corresponding signaling pathways (Fig. 3, C to F). Mock stimulations do not elicit any change in the BRET signal (Fig. 3A). As another control, we show that stimulation with the agonists without GPCR transfection does not elicit any signaling (fig. S5A). We also show that the expression levels of the tested Gα_o_ variants harboring the nanoluciferase tag are similar within each experiment (fig. S5B). Last, we also found that addition of the excess of antagonist restores the BRET signal back to the basal levels (fig. S5C).

With these analyses, we show that the G203R, R209C, and E246K mutants have reduced, and, importantly, varying from receptor to receptor, ability to respond to a GPCR activation. Specifically, we find that the G203R mutant displays significantly reduced capacity to transmit the D2 dopamine and M2 muscarinic signals, while signaling from the α2-adrenergic and μ-opioid receptors is comparable to that mediated by wild-type Gα_o_ (Fig. 3G). Similarly, R209C displays reduced signaling from the α2-adrenergic, D2 dopamine, and M2 muscarinic receptors and normal signaling from the μ-opioid receptors (Fig. 3G). Last, E246K is compromised in the M2 muscarinic signal transduction but not in signaling from the other three neuronal GPCRs receptors (Fig. 3G).

These findings double the number of GPCRs whose coupling to *GNAO1* mutants has been studied [previously, the coupling to D2 dopamine and α2-adrenergic receptors was studied with different methods (*11*, *23*)] and demonstrate that the three pathologic mutants are not completely incapable of activation of signaling by neuronal GPCRs. While all the three mutants display reduced (but not completely abrogated) responsiveness to the M2 muscarinic receptor activation, their signaling from the α2-adrenergic and μ-opioid receptors is only marginally or not at all reduced as compared to wild-type Gα_o_. These findings illustrate that varying degrees of defectiveness (from none to significant) in signaling from different neuronal GPCRs are attributable to different pathologic mutants. We next aimed at understanding the structural defects that underlie the distorted nucleotide handling, cellular interactions, and signaling capacities of the G203R, R209C, and E246K encephalopathy mutants.

### Displacement of the GTPase catalytic residue in *GNAO1* encephalopathy mutants in homology modeling and dynamic simulations

To gain insights into the possible structural deficiency in the G203R, R209C, and E246K encephalopathy mutants that lead to the deficient GTPase reaction and aberrant interactions with signaling partners, we performed homology modeling followed by molecular dynamics simulations of the GTP-loaded Gα_o_ wild type, and the mutants, based on the Gα_i1_-GTPγS structure (*24*). Structural analysis of the energy-minimized state reveals that R209C and E246K, mutations of the amino acids normally forming a salt bridge to fasten switch II and the α3 helix to lock Gα in the active conformation upon the GTP binding (*12*, *25*), result in a significant displacement of the Gln^205^ residue, the key to the catalytic GTPase reaction (*24*, *26*), away from the γ-phosphate (fig. S6, A, B, and D). Through molecular dynamics simulations (100 ns), we further found a significant global destabilization of Gα_o_[E246K], in agreement with a similar analysis of the Gα_i1_ structure (*12*), but not of the G203R or R209C mutants (fig. S7A and movie S1). Furthermore, analysis of the energy-minimized model of Gα_o_[G203R] reveals a somewhat different arrangement as compared to E246K and R209C mutants: Instead of acting through switch II, the substituting Arg residue engages in a direct interaction with the catalytic Gln^205^, displacing the latter from the γ-phosphate and occupying the space normally used by the hydrolysis water molecule and thus inducing its displacement (fig. S6, C and D). Despite the limitations of the modeling and molecular dynamics approaches as compared to direct structural analysis, these findings suggest a common mechanism of the loss of the GTPase reaction in the three *GNAO1* encephalopathy mutants as the displacement of the catalytic Gln^205^. The accompanying changes in Gα_o_ structure likely lead to the failure of the G protein to adopt the fully activated conformation and thus make it poorly recognizable by RGS19. Another amino acid residue, T182 playing an important role in the Gα-GTP interaction with RGS proteins (*27*, *28*), does not show a significant dislocation in the three pathologic Gα_o_ mutants (fig. S7, C to F).

### High-throughput assay aiming at recovering the GTPase activity of Gα_o_[E246K] identified zinc pyrithione as a drug specifically acting on the mutant but not wild-type proteins

**Fig. 4.**
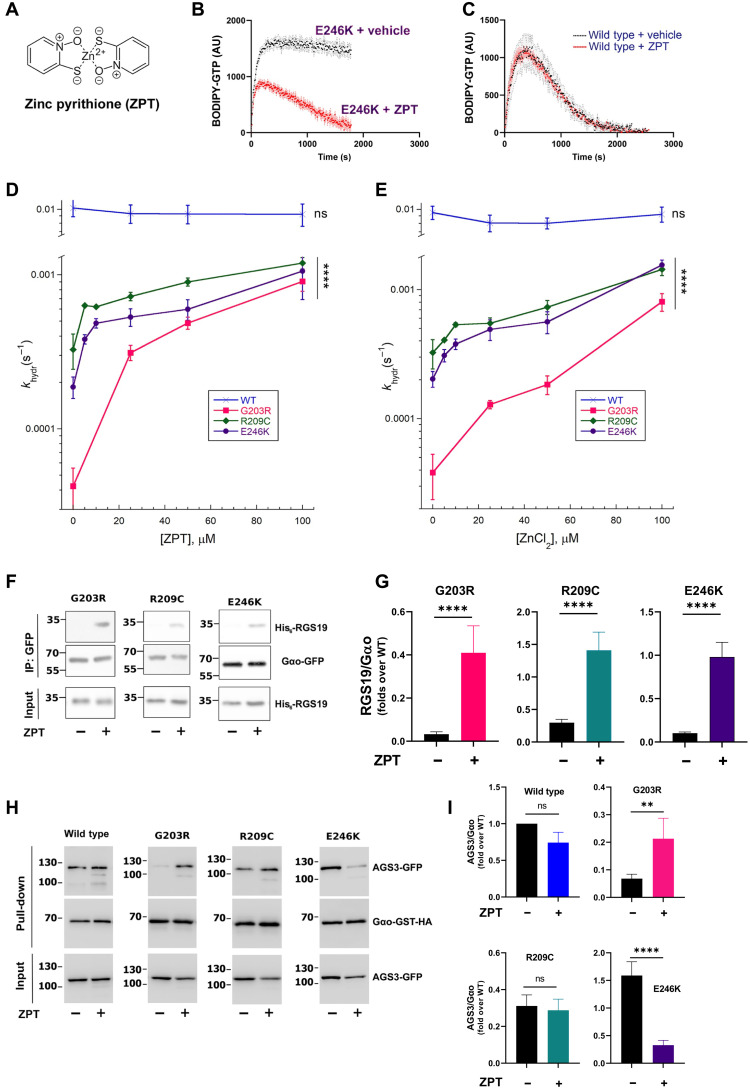
ZPT and Zn^2+^ restore GTPase activity and cellular interactions of pathologic Gα_o_ mutants. (**A** to **C**) ZPT (A) restores GTPase activity of Gα_o_[E246K] (B), not affecting GTP binding and hydrolysis of WT Gα_o_ (C). Representative curves of BODIPY-labeled GTP binding and hydrolysis are provided. (**D** and **E**) Quantification of *k*_hydr_ of Gα_o_ WT, Gα_o_[G203R], Gα_o_[R209C], and Gα_o_[E246K] treated with ZPT (D) and ZnCl_2_ (E). Note the log scale in *y* axes. (**F** and **G**) N2a cells were cotransfected with His_6_-RGS19 and Gα_o_-GFP (C-terminally tagged) variants. The next day, cells were treated with dimethyl sulfoxide (DMSO) or 1 μM ZPT for 3 hours before IP of Gα_o_ with nanobody against GFP; coprecipitation of RGS19 was analyzed by SDS-PAGE and Western blot. Abs against GFP and His_6_-tag were used to detect Gα_o_ and RGS19, respectively (F). Quantification of co-IP of His_6_-RGS19 by Gα_o_ variants, normalized to the binding levels of WT Gα_o_ (G). (**H** and **I**) Cotransfection with AGS3-GFP and Gα_o_-GST-HA (C-terminally tagged) variants was similarly followed by treatment with DMSO or 1 μM ZPT before Gα_o_ pull-down with glutathione beads. Ab against HA was used to detect Gα_o_ and against GFP to detect AGS3 (H). Quantification of the coprecipitation of AGS3-GFP by Gα_o_-GST-HA variants normalized to the binding levels of WT Gα_o_ (I). Data in (B) to (E), (G), and (I) are means of ≥3 biological replicates ± SEM; the means in (B) and (C) are shown as dots, and SEM deviations are shown as the error bars (black for no drug; red for ZPT). (D and E) ****P < 0.0005; ns, not significant by one-way ANOVA to verify the significance of the concentration dependence (as significance of the mean change upon changing concentrations of ZPT/ZnCl_2_). (G and I) **P < 0.01; ****P < 0.0001 on log-transformed data by two-way ANOVA with Sidak corrections for multiple comparisons.

We next argued that since the inability of the three Gα_o_ encephalopathy mutant proteins to hydrolyze GTP represents an easily measurable biochemical characteristic, one could design an assay to screen for molecules potentially capable of correcting this deficiency. To build such a high-throughput screening (HTS) platform, we used Gα_o_[E246K] and BODIPY-GTP to monitor the GTP uptake and hydrolysis by the mutant, as described in the first section, and screened a library of 2736 U.S. Food and Drug Administration (FDA)–approved and pharmacopeial drugs (fig. S8A). While wild-type Gα_o_ hydrolyzes all BODIPY-GTP provided to it in this biochemical setting within 10 min, Gα_o_[E246K] fails to do so, resulting in stable BODIPY fluorescence (see Fig. 1D). Thus, the initial screening was based on the identification of drugs capable of inducing a drop in fluorescence by the 10-min incubation of Gα_o_[E246K] with BODIPY-GTP. The screening was followed by hit validation, which resulted in three hit compounds: sennoside A, sennoside B, and zinc pyrithione (ZPT; Fig. 4, A and B, and fig. S8, B to E). Of those, sennosides A and B were found to decrease GTP uptake by Gα_o_[E246K] rather than GTP hydrolysis by it (fig. S8, D and E). In contrast, ZPT partially restored the GTP hydrolysis (Fig. 4B). When retesting the drugs on wild-type Gα_o_, sennosides were found to equally affect the GTP uptake by it, as seen by a decrease in the peak of BODIPY fluorescence upon treatment of wild-type Gα_o_ with the compounds (fig. S8, F and G). In contrast, ZPT produced no effect on the GTP uptake and hydrolysis by the wild-type Gα_o_ (Fig. 4C), revealing the specificity toward Gα_o_[E246K] and immediately raising our interest to this molecule.

### Zn^2+^ is the active component of ZPT restoring GTPase activity of the three encephalopathy Gα_o_ mutants in vitro

We next found that ZPT revealed a concentration-dependent restoration of the GTPase activity of all the three encephalopathy Gα_o_ mutants that we studied: G203R, R209C, and E246K (Fig. 4D and fig. S9, A to C). ZPT is a coordinated complex of pyrithione, a membrane-permeable ionophore (*29*), and Zn^2+^ (Fig. 4A) and has the primary indication to treat dandruff and seborrheic dermatitis (*30*); other biological activities of ZPT including antiviral and anticancer have also been reported (*31*, *32*). Zn^2+^ ions are poorly penetrant through cellular membranes (*33*), and the pyrithione moiety of the drug serves to deliver the ions inside cells (*34*). To test whether Zn^2+^ ions are the active component of ZPT in restoring the GTPase activity, we applied 1 mM EGTA, an efficient chelator of Zn^2+^ but not Mg^2+^ (*35*), the latter present in our experiments at the concentration of 10 mM (see Materials and Methods). As shown in fig. S9 (D and D′), zinc chelation by EGTA abolishes the restoration of the GTPase activity on Gα_o_[G203R]. We next directly tested the two components of ZPT, Zn^2+^ and pyrithione, for their ability to restore the GTPase reaction, finding that ZnCl_2_, in an EGTA-sensitive manner, was active in restoring the GTPase activity unlike the “empty” ionophore (fig. S9, D, D′, E, and E′). We further tested several other metal ions, revealing that none of them recapitulated the effect of Zn^2+^: Co^2+^, Fe^2+^, Ni^2+^, Mn^2+^, and Li^+^ were inactive in restoring the GTPase reaction, while Cu^2+^ at 100 μM appeared to completely inactivate the G protein (fig. S9, F and F′). Last, concentration dependence analysis showed that ZnCl_2_ was similar to ZPT in restoration of the GTPase activity in the three encephalopathy Gα_o_ mutants (Fig. 4E and fig. S9, G to I). In contrast, the effect of ZPT or ZnCl_2_ on wild-type Gα_o_ was confirmed to be insignificant (Fig. 4, D and E).

### Potential mechanism of action of Zn^2+^ in restoring the GTPase activity of *GNAO1* encephalopathy mutants suggested by homology modeling and dynamic simulations

We applied structural modeling and molecular dynamics simulations to gain insights into the potential mechanism of action of Zn^2+^ in the restoration of the GTPase activity of the three Gα_o_ mutants. We argued that Zn^2+^ can replace Mg^2+^ in the Gα_o_’s active center upon the interaction with GTP: It is well known that Mg^2+^-binding sites of proteins can generally be substituted by a broad range of other divalent metal ions, with Zn^2+^ being one of the most potent substitutes (*36*). We used the CHARMM36m field in dynamic simulations that provides distinct parameters for the Mg^2+^ and Zn^2+^ ions (see Materials and Methods), permitting comparative assessment of their effects. Our analysis shows that the substitution of Zn^2+^ for Mg^2+^ in the active center does not affect the global structure in the energy-minimized state of wild-type Gα_o_ (fig. S7B and movie S2). The global rearrangement observed in Gα_o_[E246K] was, to a certain degree, further aggravated in the Zn^2+^-bound protein; no global effect of Zn^2+^, however, was seen for the G203R and R209C mutants (fig. S7B and movie S2), suggesting that it is unlikely to represent the general or main mechanism of Zn^2+^ action to restore the GTPase activity.

**Fig. 5.**
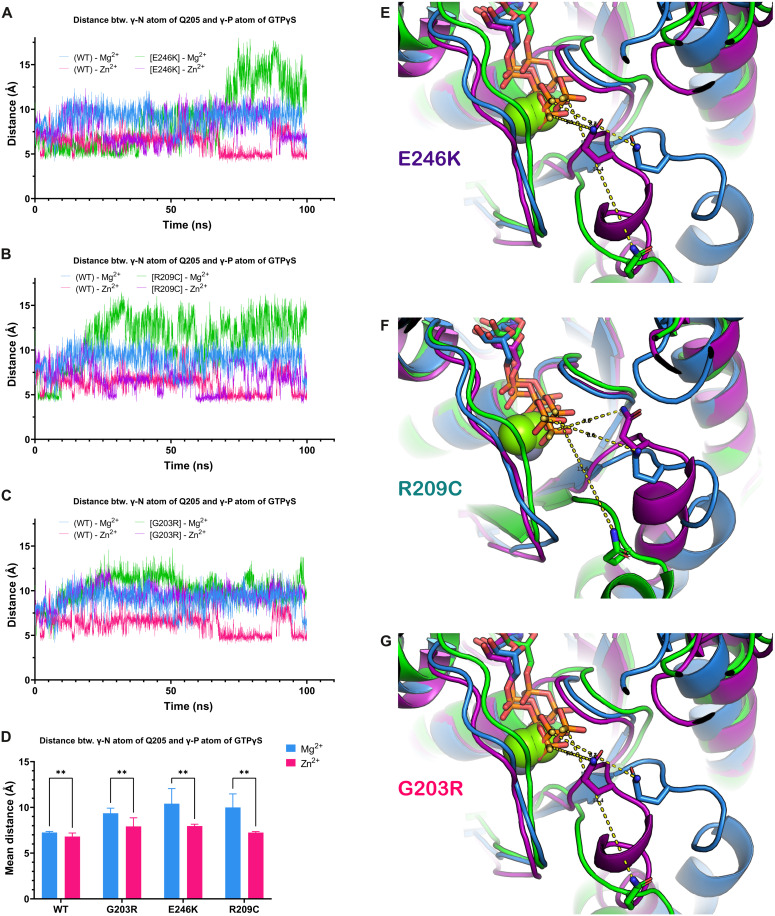
Zn^2+^ restores the defects induced to the catalytic Q205 by the pathologic Gα_o_ mutations. (**A** to **D**) Analysis of the distance between the γ-N atom of Q205 residue in Gα_o_ and the γ-P atom of GTP in WT and mutant proteins over 100 ns of molecular dynamics simulation. The panels show the plots of the measured distance between these atoms in WT protein containing Mg^2+^ or Zn^2+^ and the same in the [E246K] mutant (A), [R209C] mutant (B), and the [G203R] mutant (C). The graphs demonstrate that, over the major or significant part of the simulation trajectories in all three mutants, the Q205 residue is further away from the active site than in WT protein, which is rescued by the presence of Zn^2+^ in the active site. (A to C) Individual MD simulations. (D) Summary of the last 50 ns of three independent MD simulations presented as means ± SD. Statistical analysis: Two-way ANOVA followed by Fisher’s least significant difference multiple comparisons test; **P < 0.01. (**E** to **G**) Three-dimensional structures of the representative states with the maximal removal of Q205 from the active site are shown for the WT in the Mg^2+^-bound conformations versus the mutants in the Mg^2+^- and Zn^2+^-bound conformations: [E246K] (E), [R209C] (F), and [G203R] (G). It is evident that, upon Zn^2+^ binding, the mutants’ Q205 is brought back to the γ-P atom of GTP (the hydrogen bonds are indicated). Color coding as in the respective (A) to (C) panels.

We thus next paid special attention to the position and flexibility of the catalytic Gln^205^ that we found to be displaced from the GTP’s γ-phosphate in the pathologic mutants (see fig. S6). Our molecular dynamics simulations reveal that, for all three mutant variants, distance between the γ-N atom of Gln^205^ and the γ-P atom of GTP (increased as compared to the wild-type Gα_o_) is reduced back to the wild-type levels by Zn^2+^ (Fig. 5). Note that in the wild-type protein, Zn^2+^ also decreases the distance between Q205 and γ-phosphate but to a considerably smaller extent that has no further influence on the catalytic activity (Fig. 5, A to D). This effect is particularly evident for the Gα_o_[E246K] and Gα_o_[R209C] mutants (Fig. 5, A, B, and D to F), agreeing well with the fact that the residues E246 and R209 normally form the salt bridge to fasten the active conformation upon the GTP binding (*12*, *25*). The effect of Zn^2+^ on the distance between Gln^205^ of Gα_o_[G203R] and the γ-phosphate was also clear (Fig. 5, C, D, and G). We also measured the dynamics of the bond, as the distance between the γ-N atom of Gln^205^ and the ζ-atom of Arg^203^, in the Mg^2+^-bound versus Zn^2+^-bound conformations. This new Arg in position 203 forms a hydrogen bond with Gln^205^, and this might contribute to the loss of the GTPase activity in the mutant [see fig. S6 (C and D)]. Unexpectedly, we found that Zn^2+^, if anything, stabilized the Arg^203^-Gln^205^ interaction (fig. S7, G and H).

Overall, our structural modeling and molecular dynamics analysis suggests the atomistic mechanism for the action of Zn^2+^ on the restoration of the GTPase activity of the E246K, R209C, and G203R mutants as the bringing back of the catalytic Gln^205^ to the γ-phosphate of GTP, otherwise swayed away by the mutations. More complex modeling (e.g., through quantum mechanics or hybrid quantum/molecular mechanics, taking into consideration differences in coordination sphere configurations of Zn^2+^ and Mg^2+^) and biophysical measurements will further advance these initial findings.

### ZPT restores cellular RGS19 interactions of *GNAO1* encephalopathy mutants

Our in vitro data show that ZPT and its ion component, Zn^2+^, are able to restore the GTPase activity of the pathologic Gα_o_. We next wondered whether such a restoration could be seen in cells and be reflected in restored interactions with Gα_o_ partners. We first tested whether the mutants’ interaction with RGS19 can be restored in the N2a cells. It is however known that, acutely, Zn^2+^ can cross the cellular membranes either by active transporters (*33*) or with the help of ionophores such as pyrithione (*34*). On the other hand, a significant neuronal and other cell toxicity of ZPT has been reported, mainly because of its effectiveness in bringing large concentrations of zinc inside the cells (*32*, *37*, *38*). Thus, we first investigated the cytotoxicity of ZPT, pyrithione, and ZnCl_2_ in N2a cells. This analysis confirms the neurotoxicity of ZPT with the half-maximal inhibitory concentration of ca. 5 μM; ZnCl_2_, in contrast, was not toxic up to the concentrations of 100 μM (fig. S10A), as, presumably, it failed to penetrate the cells in this acute setting. For the subsequent experiments on the restoration of the Gα_o_-RGS19 interactions in N2a cells, we took the highest tested agents’ concentrations that did not display any cytotoxicity: 1 μM for ZPT and 100 μM for ZnCl_2_.

Using these concentrations, we found that ZPT could recover the ability of the G203R, R209C, and E246K mutants to interact with RGS19 (Fig. 4, F and G); ZnCl_2_, in contrast, was ineffective (fig. S10, B and C). The interaction of wild-type Gα_o_ with RGS19 appeared to increase by ZPT ca. twofold (fig. S10, B and C), a modest increase as compared to the effect of ZPT on the mutant Gα_o_ versions: ca. 6-fold for Gα_o_[R209C], ca. 12-fold for Gα_o_[E246K], and 20-fold for Gα_o_[G203R], recovering the interactions with RGS19 to levels of the wild-type protein (Fig. 4, F and G, and fig. S10, B and C).

We similarly tested whether ZPT could correct the aberrant cellular interactions of the Gα_o_ mutants with another partner, AGS3. As we showed above (Fig. 2, D and E), Gα_o_[G203R] and Gα_o_[R209C] demonstrate decreased interactions and Gα_o_[E246K] increased interactions with AGS3 as compared to the wild-type protein. We found that cell treatment with ZPT tends to normalize these interactions, increasing the binding of AGS3 to the G203R mutant and decreasing to the E246K mutant (Fig. 4, H and I). Last, we tested the effect of ZPT on the most aberrant, among the three pathologic Gα_o_ mutants, interaction with Gβγ, the twofold increased binding by Gα_o_[E246K] (Fig. 2C), finding a correction to the wild-type levels of the Gα_o_[E246K]-Gβγ binding by the drug (fig. S10, D and E).

Thus, we conclude that cell supplementation with Zn^2+^ ions restores the normal conformation on the three pathologic mutants of Gα_o_, bringing to the wild-type level the G protein interactions with RGS19, the partner preferring the GTP-bound form of Gα_o_, as well as with AGS3 and even Gβγ preferring the GDP-bound state of Gα_o_. These recovery effectiveness in cells prompted us to next ask whether zinc can be effective at a higher level of complexity, i.e., in an organism model of *GNAO1* encephalopathy.

### Zn^2+^ dietary supplementation rescues the *Drosophila *model of *GNAO1* encephalopathy

**Fig. 6.**
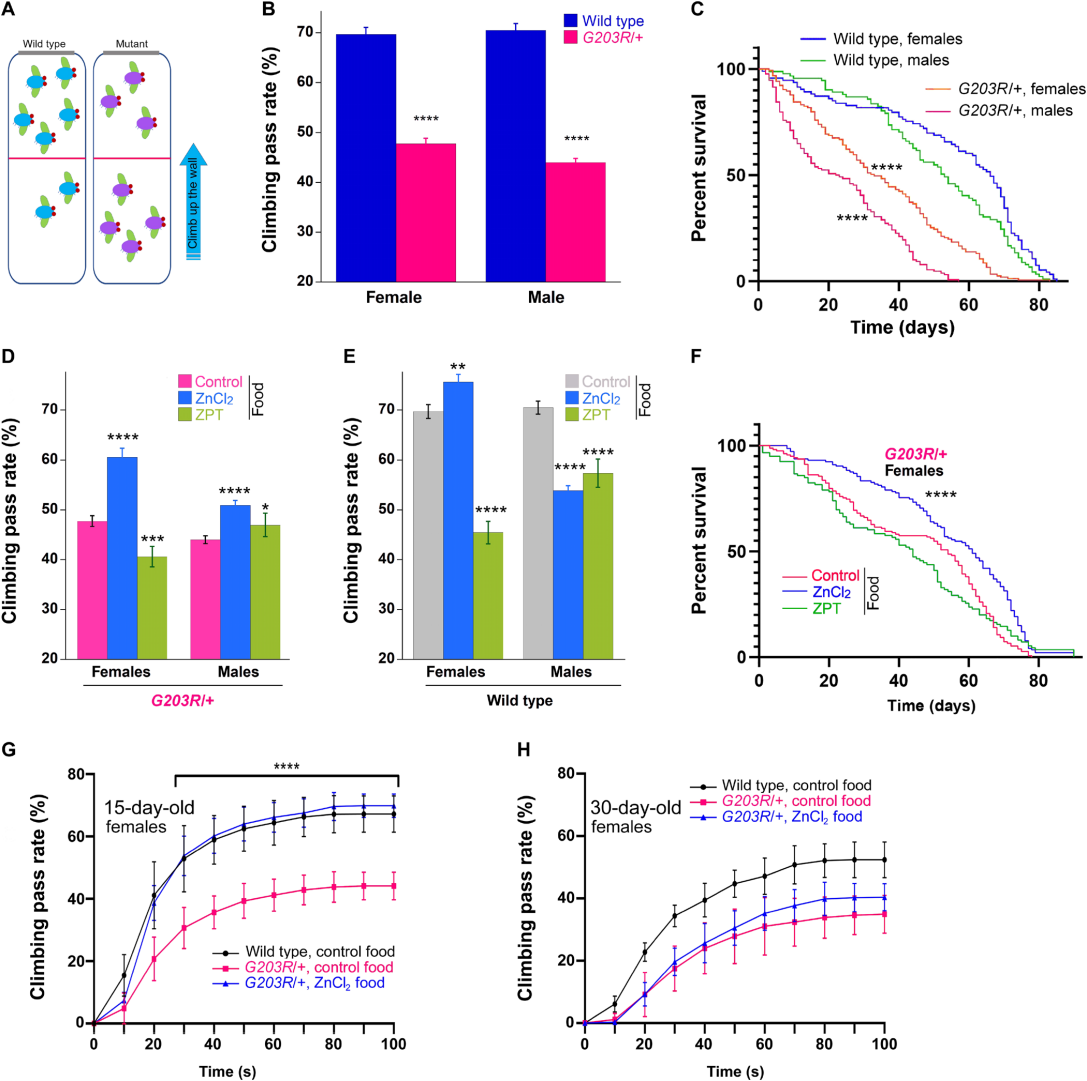
Dietary Zn^2+^ rescues motor dysfunction and life span in *Drosophila* model of *GNAO1* encephalopathy. (**A**) Negative geotaxis: After tapping down, flies climb up the vial (blue arrow). The climbing pass rate is the percent of flies passing 10 cm from the bottom (red line) in 10 s. (**B**) Negative geotaxis of 100 to 110 male and female flies (in groups of ca. 15 flies). Data are means ± SEM, *n* = 10. Two-way ANOVA with Sidak corrections for multiple comparisons shows significant differences between WT and *G203R/+* flies, *P* < 0.0001. (**C**) Life span of 250 flies (110 males and 140 females) determined for 85 days. Mantel-Cox and Gehan-Breslow-Wilcoxon tests both show significant (*P* < 0.0001) drop in survival of *G203R/+* flies. Intersex life-span differences were also significant (*P* = 0.0124 for WT and *P* < 0.0001 for *G203R/+* flies, Mantel-Cox test). (**D**) Locomotion dysfunction of *G203R/+* flies rescued by dietary ZnCl_2_; ZPT shows no consistent effect. (**E**) Effects of dietary ZnCl_2_/ZPT on locomotion of WT flies. Data presentation and analysis in (D) and (E) as in (B); *P < 0.05, **P < 0.01, ***P < 0.001, and ****P < 0.0001. (**F**) Drop in life span of female *G203R/+* flies rescued by dietary ZnCl_2_. Data presentation and analysis as in (C). (**G** and **H**) WT and *G203R/+* female flies, 15 (G) and 30 (H) days old, tested in video-recorded quantitative negative geotaxis assay. Younger *G203R/+* flies show complete locomotion recovery if raised on ZnCl_2_-supplemented food (*t* test, *P* < 0.0001 for the time points starting from 30 s for the difference between control food and ZnCl_2_ supplementation). Older flies reveal only a tendency of recovery. Data are means ± SD, *n* = 7 to 10. Age-dependent decrease in the climbing rate for WT [black curves in (G) and (H)] and *G203R/+* flies [red curves in (G) and (H)] is highly significant (*P* < 0.0001 for both WT and mutant flies).

Animal models have the instrumental role in deciphering the human disease mechanisms and in identifying/validating the treatment routes. The fruit fly *Drosophila melanogaster* represents an excellent model organism for studies in various fields of biology (*39*), and our recent work highlights the power of *Drosophila* as the host to model *GNAO1* encephalopathy (*40*). To establish a *GNAO1* encephalopathy model in the fruit fly, we applied the CRISPR-Cas9 mutagenesis together with phiC31-mediated recombinase-mediated cassette exchange (RMCE) (*41*) to introduce the pathogenic G203R mutation into the *Drosophila* Gα_o_ (see Materials and Methods and fig. S11). We found that the resultant *[G203R]/[G203R]* flies are second larval stage lethal [as compared to the late embryonic lethality of homozygous *G*α*o* null mutants (*42*, *43*)], while *[G203R]/+* flies are viable and fertile yet reveal a number of deficiencies. Specifically, the heterozygous mutant flies manifest a significant motor dysfunction, measured in the negative geotaxis assay as a reduced capacity to climb up the wall (Fig. 6, A and B), reminiscent of the motor dysfunction in the human patients. Furthermore, the encephalopathy mutant *Drosophila* displays a twofold reduction in the life span (Fig. 6C). Regional brain atrophy, sometimes progressive, has been described in *GNAO1* patients with the G203R mutation and may be the outcome of epileptic onsets (*6*, *44*–*46*). Analysis of 35-day-old *[G203R]/+* flies revealed limited yet significant brain degeneration (fig. S12). However, this degeneration was very modest as compared to that observed, e.g., in *Drosophila* models of Alzheimer’s disease (*47*), the finding that may be aligned with the fact that the *G203R* mutant flies did not display any signs of spontaneous epilepsy. Given the neonatal lethality of *GNAO1[G203R]/+* mice (*9*), we thus establish the first viable animal model of G203R encephalopathy with this *Drosophila* line capable of recapitulating some of the clinical manifestations of the disease.

Dietary zinc supplementation has been applied to treat various human health conditions, including the neurological ones such as depression (*48*), epilepsy (*49*), psychiatric and neurodegenerative diseases (*50*), or sleep disorders (*51*), as well as to support normal neonatal development (*52*). We thus wondered whether dietary supplementation of zinc in the *Drosophila* model of *GNAO1* encephalopathy may reveal any beneficial effects. Although, in the acute setting, we could not observe an effect of ZnCl_2_ to N2a cells (fig. S10), we argued that the continuous supplementation through diet could make a difference. Food supplementation of ZnCl_2_ to the final concentration of 200 μM has been previously shown to rescue the survival of *Drosophila* mutant for *dZip1* and *dZip2*, the gut zinc transporters (*53*), providing us a guideline. We also tested 10 μM ZPT food supplementation, following the application of up to 15 μM ZPT in rats (*54*). *Drosophila* lines, *G203R/+* and the wild-type control (see Materials and Methods and fig. S11), were raised from the egg at the standard food or that supplemented with ZnCl_2_ or ZPT. The climbing capacities of the resultant populations were compared, along with their longevities.

We found that the ZnCl_2_-containing food significantly improves the motor function of the *G203R/+* flies; ZPT did not show consistent effects (Fig. 6D). The effect of ZnCl_2_ was particularly strong for female *Drosophila*, bringing the climbing capacity toward the wild-type levels. The control wild-type flies showed sex-variable effects: slight improvement for females and decrease for males (Fig. 6E); ZPT was decreasing the climbing rate for both sexes, likely reflecting its toxicity upon systemic administration (*54*). ZnCl_2_ food supplementation also rescued the reduced life span of female *G203R/+* flies (Fig. 6F); no effect could however be seen for ZPT or for male flies (Fig. 6F and fig. S13A).

To gain more insights into the motor dysfunction of the *G203R/+* flies and its restoration by the dietary zinc, placed in the context of the animal aging, we applied a modified, more detailed negative geotaxis assay (*55*) that permits to quantify the locomotion over different distances and time frames (see movie S3). With this analysis, comparing wild-type and *G203R/+* flies of the age of 15 and 30 days, we see a progressive age-dependent decline in the climbing capacity of both genotypes (Fig. 6, G and H). Younger female *G203R/+* flies reveal a complete recovery of their climbing capacity upon the ZnCl_2_ dietary supplementation (Fig. 6G and movie S3), while ZPT was ineffective. Less pronounced but significant effects were also seen in males, where dietary ZPT had a similar effect (fig. S13B). The ability of dietary ZnCl_2_ to restore the mutant fly locomotion declined with age (Fig. 6H and fig. S13, C and D).

Overall, these observations reveal strong improvements of the behavioral and life span conditions in the *Drosophila* model of *GNAO1* encephalopathy by dietary zinc supplementation. Given the large body of clinical evidence on the dietary zinc supplements for human patients with diverse neurological conditions, our findings may speak for the applicability of such supplementation for the patients with pediatric *GNAO1* encephalopathy. These issues and the sex- and age-sensitive effects of the dietary zinc supplementation are discussed in the next section in the context of the probable need for the continuous dietary zinc supplementation to produce therapeutic effects.

## DISCUSSION

*GNAO1*-dependent pediatric encephalopathy is a recently diagnosed rare yet devastating neurological disease. The number of found different, mostly missense point, mutations in *GNAO1* causing this malady steadily increases every year since the first 2013 report (*5*). However, despite some insights (*5*, *9*, *19*, *23*, *56*, *57*), the understanding of the molecular etiology underlying the pathological developments has been largely missing. This delay in the understanding blocks development of therapies to treat the patients. Being mostly unresponsive to the conventional anti-epileptic treatments, the sick children have so far demonstrated the best, albeit partial, response to the symptomatic, highly invasive and poorly accessible therapy: deep-brain stimulation (*13*).

The molecular dysfunction that we have “diagnosed” here for the three most common *GNAO1* encephalopathy mutations is the constitutive GTP-binding state of the mutant Gα_o_ proteins, resulting from a strongly increased rate of GTP uptake concomitant with a markedly reduced rate of GTP hydrolysis. Molecular dynamics and structural modeling provide us with the likely unifying mechanism of this dysfunction: Each of the three mutations induces a displacement of the catalytic Gln^205^; this change is also likely the reason for the defective interaction of the three mutants with RGS19. We have further observed abnormal cellular interactions with some other Gα_o_ partners, such as Gβγ and AGS3, which interact less with Gα_o_[G203R] and Gα_o_[R209C] but unexpectedly more with Gα_o_[E246K] than with wild-type Gα_o_. In contrast, interactions with the Golgi partners of Gα_o_ are near normal in the pathologic mutants: the findings that, together with the decreased response of the mutants to neuronal GPCRs, suggest that the three encephalopathy mutations that we studied here affect rather the plasma membrane than the Golgi pools of Gα_o_-mediated activities (*16*, *19*).

The following considerations are needed before interpreting the complex cellular interaction aberrations of the pathologic Gα_o_ variants. Long-lived pools of monomeric GDP-loaded Gα_o_ are induced by GPCRs (*58*), and many signaling partners of Gα_o_ do not discriminate between GTP- and GDP-bound states of the protein (*16*, *59*–*61*). In contrast, regulators of Gα_o_ activities “care” about the nucleotide state of the G protein: Gβγ and AGS3 act as guanine nucleotide dissociation inhibitor proteins that bind the GDP form of Gα_o_ (*2*, *17*, *21*, *62*), and RGS19 acts as a GTPase activating protein that recognizes the GTP form (*3*, *4*). Put together with our biochemical and cellular characterizations of the pathologic Gα_o_ mutants, these considerations may speak for the following. First, given the lack of cellular interaction with the RGS protein whose function is to speed up GTP hydrolysis (*3*, *4*), the cellular GTP residence of the mutants is expected to be even more pronounced, which could be one possible explanation for the reduced interactions of Gα_o_[G203R] and [R209C] (but not of [E246K]) with Gβγ and AGS3. Second, despite the inferred increased residence in the GTP-bound state, the mutant Gα_o_ proteins likely do not adopt the truly activated conformation and hence fail to interact with RGS19. Third, the abnormal interactions with Gβγ and AGS3 (with the paradoxical increase of this interaction for Gα_o_[E246K]) might indicate that the pathological mutants have aberrated conformations not only in their GTP-bound state but also when GDP-bound. As Mg^2+^ was found to occupy the same location in the catalytic site of Gα_i1_ in both nucleotide states (*63*), its substitution with Zn^2+^ could be expected to influence both GTP and GDP forms of Gα_o_. In agreement, we find that cell treatment with ZPT not only recovers the RGS19 interactions of the three pathologic mutants but also tends to correct their interactions with AGS3: up for the G203R and down for the E246K. The aberrant E246K-Gβγ interactions are also corrected by ZPT. More work may be needed to gain structural insights into the GDP-GTP conformations and dynamics of the Gα_o_ mutants and the effect of zinc ions on them.

The constitutive GTP binding by the G203R, R209C, and E246K mutant forms of Gα_o_ suggests the gain-of-function (GOF) nature of these mutations: hypermorph in the classical Muller classification of gene mutations (*64*). This observation is consistent with the findings that Gα_o_[R209C] induced higher levels of Akt and S6 kinase phosphorylation than the wild-type protein in white blood cells, stimulating cell proliferation and neoplastic transformation, ultimately contributing to childhood acute lymphoblastic leukemia (*65*). Similarly, Gα_o_[G203R] and Gα_o_[E246K] have been delineated as GOF mutants on the basis of their ability to decrease the median effective concentration values for α_2A_ adrenergic receptor-mediated inhibition of cyclic adenosine monophosphate as compared to wild-type Gα_o_ (*11*).

However, we find that, despite GTP binding, the three mutants fail to adopt the proper activated conformation, resulting in the aberrant interaction with Gα_o_ partner proteins. Furthermore, we find that G203R, R209C, and E246K have a reduced potential to respond to a panel of neuronal GPCRs. These features rather speak for a loss-of-function (LOF) or a partial LOF nature of the mutants, amorph and hypomorph, respectively (*64*), which is the conclusion that agrees with some other studies on these *GNAO1* mutations (*23*, *56*). Some features of the *Drosophila* model of the disease, namely, the delayed lethality of the *G*α*o[G203R]/G*α*o[G203R]* mutant flies as compared to homozygous *G*α*o* null mutants (*42*, *43*), also speak in favor of the hypomorph nature of the mutation. Last, from human patients to the *Drosophila G*α*o[G203R]/+* model, agreeing also with mouse (*9*, *23*) and nematode (*56*, *57*) models of the disease, and further agreeing with some in vitro observations (*23*), the encephalopathy *GNAO1* mutations have been described in many instances as dominant negative or antimorph in the Muller classification (*64*).

This multitude of ways to genetically ascribe the nature of the encephalopathy *GNAO1* mutations has created a significant confusion and debate in the field. Individual amino acid changes may have counteracting effects on different biochemical properties of the G protein (nucleotide binding and hydrolysis, interaction with regulators and effectors, structure of the active state, etc.), resulting in the emergence of the pathogenic mutations as hypermorph, amorph/hypomorph, or antimorph depending on the specific biological property that is considered. Alternatively, these mutations might be described using the only remaining type within the Muller classification, the neomorph. As described in Muller’s classical genetics work (*64*), neomorph represents a “change in the nature of the gene at the original locus, giving an effect not produced, or at least not produced to an appreciable extent, by the original normal gene.” In cancer, a large number of oncogenic mutations in different genes, previously considered GOF or LOF, now emerge as neomorphs (*66*). The recognition of a given disease-causing mutation as neomorphic imposes important restrains on the drug discovery efforts, as mere increase in the normal (nonmutated) gene function or pharmacological modulation of the function of the wild-type allele cannot be efficient in counterbalancing the neomorphic pathological function (*64*, *66*). Instead, drug discovery efforts should be dedicated directly to the aberrant neomorphic activity, ideally (for the sake of minimizing side effects) not affecting the wild-type protein.

It is in this paradigm that we designed our drug discovery aiming at recovering the pathologic, biochemically measurable function of mutant Gα_o_, in the counter screen testing the drug candidates against wild-type Gα_o_. It is remarkable that a simple ion, Zn^2+^ emerging from our screening of drug candidates to recover the GTPase deficiency of the mutant Gα_o_, is efficient in restoring the structural rearrangements induced by the pathologic mutations with a minimal action on the wild-type protein. Our initial molecular mechanics modeling, albeit simplistic, suggested that, replacing Mg^2+^ from the GTP pocket of Gα_o_, Zn^2+^ has a tendency to bring back the catalytic Gln^205^ to the vicinity of the γ-phosphate of GTP. This trend in the structural rearrangements likely reflects the mechanism behind the ability of Zn^2+^ to recover the GTPase reaction of the three pathologic mutants, their cellular interactions with RGS19 and AGS3, and ultimately provide motor activity and longevity ameliorations in the *Drosophila* model of *GNAO1* encephalopathy upon dietary zinc supplementation. In the future, more quantitative and in-depth insights into the effects of Mg^2+^ substitution by Zn^2+^, by modeling and experiments, will deepen the molecular understanding of this phenomenon.

Dietary zinc supplements have found numerous applications in human health. Being safe, they have been shown to improve neonatal brain development (*52*) and sleep quality in adults (*51*) and to ameliorate health conditions in depression (*48*), epilepsy (*49*), and a set psychiatric and neurodegenerative states (*50*). For example, daily 25 mg of Zn^2+^ applied for 6 weeks in one study (*67*), as well as daily 220 mg of zinc sulfate (providing 50 mg of zinc) applied for 12 days in another (*68*), was found to positively act on patients with depression. The upper limits of dietary zinc with no observed adverse effects, as set by the World Health Organization, are 13 mg/day for the age of 7 to 12 months, 23 mg/day for the age of 1 to 6 years, and 45 mg/day for adults (*69*). In this regard, dietary zinc supplementation might be considered as a potential treatment option to improve the conditions of patients with *GNAO1* encephalopathy, at least those carrying the G203R, R209C, and E246K mutations. More studies will show how applicable is the Zn^2+^-restorable GTPase deficiency mechanism to the other *GNAO1* encephalopathy mutants.

Despite decades of diverse applications of zinc supplements in human health, the details of bioavailability, pharmacokinetics, pharmacodynamics, and potential toxicities of the dietary zinc are still controversial (*50*, *70*). Multiple factors confound the efficiency of zinc absorption from the gut, its penetration through the blood-brain barrier, and the ultimate entry and activities within neuronal cells (*50*, *71*, *72*). For example, the different efficiency of dietary zinc supplementation in rescuing the motor dysfunction and reduced life span in female versus male fruit flies might be related to different expression of certain zinc transporters in the two sexes (*73*). Alternatively, higher food consumption by female *Drosophila* [by ca. threefold over that in males given the need of egg production (*74*, *75*)] possibly results in higher accumulation of dietary zinc, thus contributing to the better responses. We find using the *Drosophila* model that the rescuing capacity of the dietary zinc decreases with age. Adult *Drosophila* consumes much less food than larvae, and a further marked decline in food consumption in adults after the age of 2 weeks has been observed (*74*), thus possibly contributing to the decreased performance of aging *G203R/+* flies despite the availability of the zinc-supplemented diet. Collectively, these observations might speak for necessity of continuous dietary supplementation to maintain the healing effects in patients.

Pyrithione is a chelator bringing zinc ions across cell membranes (*29*, *34*), hence the ability of ZPT to restore the mutant Gα_o_ functions in cell cultures. However, given the toxicity of ZPT upon systemic administration (*54*) also seen in our *Drosophila* experiments, its indications as a drug are restricted to topical applications to treat dandruff and seborrheic dermatitis (*30*). Diverse approaches to enhance and control safe zinc uptake and delivery are being developed, from nutritional chelators and nanoparticle carriers to intravenous, cerebrospinal, or intrabrain injections (*50*, *72*, *76*), and might be considered in the future prospective applications to patients with *GNAO1* encephalopathy. As a possible alternative to the dietary supplementation, intravenous zinc (as sulfate, chloride, or gluconate salts) is part of the parenteral nutrition protocols, with the dosages of 400 μg/kg per day recommended for premature infants, 250 μg/kg per day for term infants below 3 months, and 100 μg/kg per day for children above 3 months of age; adult dosages vary from 2.5 to 30 mg/day (*77*).

To sum up, the study presented here sweeps from the understanding, at the molecular and even atomistic level, of the core biochemical dysfunctions seen in the three most frequent *GNAO1* encephalopathy mutations to assay establishment and screening for drug candidates to rescue this dysfunction, followed by the candidate validation in biochemical and cellular models. Last, we establish an animal model of *GNAO1* encephalopathy and show that a dietary supplementation of Zn^2+^, the active component of the treatment, provides a significant rescue of the movement disorder and life-span shortening of the mutant animals. Our work sheds light on the basic functions of the major neuronal G protein and on the molecular etiology of *GNAO1* encephalopathy and might serve as grounds for recommendation of the dietary zinc supplementation as a treatment option for patients with *GNAO1* encephalopathy.

## MATERIALS AND METHODS

### Plasmids and molecular cloning

The plasmids for the Gα_o_-GFP (C-terminally and internally tagged), Gα_o_-GST-HA (C-terminally tagged), monomeric RFP (mRFP)–Gβ1, mRFP-Gγ3, His_6_-RGS19, GFP-Rab3a, and AGS3-GFP were previously described (*4*, *14*, *16*, *78*, *79*). Arf1-mRFP was generated by replacing the Age I/Not I GFP sequence of Arf1-GFP (*16*) with the corresponding mRFP sequence from the mRFP-N1 plasmid (*16*). KDELR-HA (hemagglutinin) was obtained by replacing the Age I/Not I GFP sequence of KDELR-GFP (*16*) with three consecutive HA tags generated by aligned oligonucleotides (table S1). The Gα_o_ mutants were obtained by site-directed mutagenesis in the pcDNA3.1 plasmid (*4*) using the primers as listed in table S1. The plasmid pET-23b encoding wild-type N-terminally tagged 6xHis-Gα_o_ (*4*) was used to create E246K, R209C, and G203R mutants through subcloning using restriction sites Sph I and Eco RI from the constructs in pcDNA3.1.

### Protein production and purification

The Rosetta-gami *Escherichia coli* strain was transformed with pET23b-Gα_o_ wild type, pET23b-Gα_o_[G203R], pET23b-Gα_o_[R209C], or pET23b-Gα_o_[E246K] and grown at 37°C to an optical density at 600 nm of 0.6 before induction with 1 mM isopropyl-β-d-thiogalactopyranoside and additional growth overnight at 18°C. Cells then were harvested by centrifugation 3500*g* at 4°C and resuspended in tris-buffered saline (TBS) [20 mM tris-HCl (pH 7.5) and 150 mM NaCl] supplemented with 1 mM phenylmethylsulfonyl fluoride (PMSF) and 30 mM imidazole. Cells were disrupted with a high-pressure cell press homogenizer; the debris was removed by centrifugation at 15,000*g* for 15 min at 4°C. The supernatant was applied to the Ni^2+^ resin (QIAGEN) overnight in a rotary shaker at 4°C. The Ni^2+^ resin was washed twice with 10 resin volumes of TBS supplemented with 10 mM imidazole. On the third wash, the washing buffer was supplemented with 3% glycerol, 10 mM MgCl_2_, 0.1 mM dithiothreitol, and 200 μM GDP. The Ni^2+^ resin was washed two more times with 10 resin volumes of the washing buffer. Proteins were then eluted with TBS containing 300 mM imidazole. To subsequently remove imidazole, the protein buffer was exchanged into TBS using Vivaspin concentrator. Protein concentration was measured using the Bradford assay, and the purity was analyzed using SDS–polyacrylamide gel electrophoresis (SDS-PAGE) followed by Coomassie staining. Gα_o_[Q205L] was purified in parallel as described (*4*).

### GTP-binding and hydrolysis assay

The GTP-binding and hydrolysis assay using BODIPY-GTP (Invitrogen) or BODIPY-GTPγS (Invitrogen) was performed as described (*4*). Gα_o_ was diluted to 1 μM in the reaction buffer [TBS supplemented with 10 mM MgCl_2_ and 0.5% bovine serum albumin (BSA)]. The mixture was then pipetted into black 384-well plates (Greiner), and BODIPY-GTP or BODIPY-GTPγS (1 μM; Invitrogen) was added into the wells. Fluorescence measurements were performed with a Tecan Infinite M200 PRO plate reader with excitation at 485 nm and emission at 530 nm at 28°C. To trace the fast kinetics of the Gα_o_[G203R] and Gα_o_[R209C], BODIPY-GTPγS was added using the injector unit followed by immediate measurement. The GTP-binding and hydrolysis data of Gα_o_–wild type were fit to obtain the *k*_bind_ and *k*_hydr_ rate constants as previously described (*4*), where the end point was set as the baseline. As Gα_o_[G203R], Gα_o_[R209C], and Gα_o_[E246K] were incapable of GTP hydrolysis, the BODIPY-GTP curve was fit with the initial fluorescence value as baseline, and *k*_hydr_ was calculated using this curve. For the BODIPY-GTPγS displacement analysis, 5 μl of 50 μM GDP solution in TBS was injected in 20 μl of the BODIPY-GTPγS–loaded Gα_o_ solution at the indicated time to the final concentration of 10 μM without interruption of measurements.

### Homology modeling and molecular dynamics analysis

The structure of wild-type GTPγS-bound Gα_o_ was homology modeled using the Protein Data Bank (PDB) 1GIA structure (*24*) on the SWISS-MODEL server with the user template setting (*80*). This structure was used as a base to generate amino acid substitutions in the PyMOL 2.4.0 software and metal ion substitutions using Check My Metal web interface (*81*). The resulting draft PDB models of Gα_o_ mutants bound to Mg^2+^ or Zn^2+^ were directly used in the GROMACS 2021.2 software (*82*, *83*) to generate both the energy-minimized models and the molecular dynamics runs. To this end, the CHARMM36m all-atom force field was used (mackerell.umaryland.edu/charmm_ff.shtml#gromacs). The structures were solvated in a cubic box with 1-nm distance from protein edges; the phosphate group charge was neutralized by Na^+^ ions. Subsequently, energy minimization and temperature and pressure equilibration were performed using typical parameters (duration, 50 ps; step, 2 fs). A 100-ns production run was performed on high-performance computation cluster of University of Geneva with 2-fs step and leap-frog integrator and with 1-nm cutoffs for van der Waals and electrostatic cutoffs. Subsequent analysis of the trajectories and structures was performed using both built-in functions of GROMACS package and PyMOL using custom scripts.

### High-throughput screening

HTS for mutant Gα_o_ modulators was performed using the Gα_o_[E246K] protein and FDA Approved & Pharmacopeial Drug Library (HY-L066, MedChemExpress). Dimethyl sulfoxide (DMSO) or compounds in DMSO (12.5 μM) were mixed with Gα_o_[E246K] at 1 μM in a reaction buffer and BODIPY-GTP at 1 μM as described in the “GTP-binding and hydrolysis assay” section above. Reaction was carried out for 10 min.

To analyze the data generated by the HTS, two parameters were calculated: (i) binding constant (*k*_bind_) and (ii) maximal GTP uptake. For candidates affecting the *k*_bind_, the hits were picked if the compound modulated *k*_bind_ by ≥2 SD of DMSO-treated wells. For candidates affecting the maximal BODIPY-GTP uptake, the hits were picked if the compound modulated the maximal GTP uptake by ≥3 SD of DMSO-treated wells. The hits were subsequently validated by performing the GTP-binding assay at 50 μM of compounds using both Gα_o_ wild type and Gα_o_[E246K]. Validations were performed using commercially available sennosides (USP), ZPT (Sigma-Aldrich), ZnCl_2_ (Sigma-Aldrich), and pyrithione (Sigma-Aldrich).

### Cell lines and culture conditions

Male mouse neuroblastoma N2a [American Type Culture Collection (ATCC), CCL-131] cells were maintained in minimum essential medium (MEM; Thermo Fisher Scientific), supplemented with 10% fetal calf serum, 2 mM l-glutamine, 1 mM pyruvate, and 1% penicillin-streptomycin at 37°C and 5% CO_2_. Human HEK293T (ATCC, CRL-3216) cells grew in Dulbecco’s modified Eagle’s medium (DMEM; Thermo Fisher Scientific), supplemented as above and under the same culture conditions.

### Immunoprecipitation and pull-down

Immunoprecipitation and glutathione *S*-transferase (GST)–based pull-down of Gα_o_-GFP and Gα_o_-GST-HA constructs was performed as previously described (*16*, *20*). Briefly, N2a cells were transfected with the constructs indicated in the corresponding figures, and after 24 hours, cells were directly harvested or incubated with fresh media supplemented with 1 μM ZPT, 100 μM ZnCl_2_, or DMSO for 3 hours at normal culture conditions. Cells were harvested with ice-cold GST lysis buffer [20 mM tris-HCl (pH 8.0), 1% Triton X-100, and 10% glycerol in phosphate-buffered saline (PBS)] supplemented with a protease inhibitor cocktail (Roche). Cell lysates were cleared by centrifugation at 16,000*g* for 15 min at 4°C. For GST-based pull-downs, supernatants were directly incubated with 20 μl of a 50% slurry of Glutathione Sepharose 4B beads (GE Healthcare) overnight on a rotary shaker at 4°C. For immunoprecipitation, cleared supernatants were incubated with 2 μg of nanobody against GFP (*84*) on ice for 30 min; then, 20 μl of Glutathione Sepharose 4B beads was added, and samples were incubated as above. Beads were repeatedly washed with lysis buffer, and bound proteins were eluted by boiling the beads with SDS-PAGE sample buffer. Samples were lastly analyzed by SDS-PAGE followed by Western blot using antibodies against GFP (dilution, 1:2000; GeneTex, GTX113617), His_6_-tag (dilution, 1:2000; QIAGEN, 34650), mRFP (dilution, 1:250; Santa Cruz Biotechnology, sc-101526), and HA-tag (dilution, 1:2000; Roche, 3F10). Peroxidase-conjugated antibodies were from Jackson ImmunoResearch (dilution, 1:20,000; 115-035-062 and 111-035-144). Quantification of blots was done using ImageJ.

### Immunofluorescence and microscopy

For microscopy, N2a cells were transfected for 7 hours, trypsinized, and seeded on poly-l-lysine–coated coverslips in complete MEM for an additional 15 hours before fixation. Cells were fixed for 20 min with 4% paraformaldehyde in PBS, were permeabilized for 1 min using ice-cold PBS supplemented with 0.1% Triton X-100, blocked for 30 min with PBS supplemented with 1% BSA, incubated with the primary antibody against GM130 (dilution, 1:500; BD Biosciences, 610823) in blocking buffer for 2 hours at room temperature, washed, and subsequently incubated with the secondary antibody and 4′,6-diamidino-2-phenylindole (DAPI) in blocking buffer for 2 hours at room temperature. The Cy3-labeled secondary antibody was from Jackson ImmunoResearch (dilution, 1:1000; 115-165-146). Coverslips were lastly mounted with VECTASHIELD on microscope slides. Cells were recorded with a Plan-Apochromat 63×/1.4 oil objective on a LSM 800 confocal microscope and further processed using the ZEN Blue software (all Zeiss). Quantification of relative localization of different variants of Gα_o_ at plasma membrane and Golgi was performed as in (*20*).

### MTT assay

N2a cells (3000 cells per well) were distributed into a transparent 384-well plate. The medium of each well was replaced by 50 μl of fresh medium the next day containing the indicated concentrations of ZPT, ZnCl_2_, or pyrithione. After incubation for 3 hours, the medium in each well was replaced by 50 μl of Thiazolyl blue (0.5 mg/ml; Carl Roth) solution in 1× PBS. The plates were incubated for 3 hours at 37°C. Then, the solution was removed, and 30 μl of DMSO was added into each well. Absorbance at 570 nm was measured in a Tecan Infinite M200 PRO plate reader.

### Analysis of the heterotrimeric G protein complex formation and dissociation by BRET

The plasmid Go1-CASE encoding nanoluciferase-tagged Gα_o_, Gβ3, and Gγ9 with the Venus tag was provided by G. Schulte (*22*). The pathologic mutations were introduced by site-directed mutagenesis using the same primers as described above to introduce corresponding mutations and the following two flanking primers 5′-AATCCAAGAGTGCTTCAACCGGTC and 5′-ATATTAACGCTTACAATTTACGCC to generate two overlapping polymerase chain reaction (PCR) fragments containing mutation for subsequent Gibson assembly in Af lII/Xma I–linearized Go1-CASE. These plasmids were cotransfected at 1:1 ratio in HEK293T cells with pcDNA3.1 as a control or the following GPCR-encoding plasmids: dopamine D2 receptor (*85*), ɑ2-adrenergic receptor, TANGO-tagged (*86*), M2 muscarinic receptor (cDNA Resource Center, #MAR0200000), and μ-opioid receptor (*87*). Twelve hours after transfection, the cells were seeded at 6000 cells per well in the transparent-bottom black 384-well plates. After an additional 24 hours, the medium was replaced by 10 μl of PBS. Furimazine was injected to 10 μM immediately before measurement, and agonist and antagonist solutions were injected sequentially at the indicated times to indicated concentrations in PBS. Reading was performed with a Tecan Infinite plate reader.

### Plasmids for *Drosophila dG*α*o* editing

#### Donor plasmid pGao47-LattP-pBacDsRed-attPR for the CRISPR-Cas9 step of transgenesis

Plasmid pHD-ScarlessDsRed (Drosophila Genomics Resource Center, Bloomington, USA, stock #1364) was modified by adding LoxP sequences after DsRed coding region, for which the annealed complementary oligonucleotides loxPfw and loxPrev (table S1) were cloned into the Not I site of this plasmid. The resultant plasmid (pScarlessDsRed-lox) was digested with Aar I and Sap I and assembled with two 110–base pair (bp) attP sequences, using the NEBuilder HiFi DNA Assembly Cloning Kit (New England Biolabs, catalog no. E5520S). Plasmid pTA-attP (Addgene, #18930) was used as a template for PCR amplifications of attP, which were performed with the primer sets attPfwRI/attPrevHpaI and attPfwKpnI/attPrevHpaI (see the list of primers below). The resultant pattP-pBacDsRed-lox-attP plasmid contains the pBac transposon with the fluorescent DsRed marker flanked with two inverted attP sequences. The left homologous arm (LHA) was PCR-amplified with the dGao47Lfw and dGao47Lrev primers from *Drosophila* genomic DNA, producing the 815-bp PCR product, which was treated with Eco RI and further cloned into the pattP-pBacDsRed-lox-attP plasmid by the Eco RI site producing the construct pLattP-pBacDsRed-lox-attP. The right homologous arm (RHA) was PCR-amplified with the dGao47Rfw and dGao47Rrev primers, and the resulting 500-bp PCR product was treated with Kpn I and then cloned into the plasmid pLattP-pBacDsRed-lox-attP digested with Kpn I and Sma I. The resultant donor plasmid pLattP-pBacDsRed-attPR contains the DsRed marker flanked with inverted attPs and the 815-bp-long LHA and 500-bp-long RHA. All PCRs were performed using Phusion High-Fidelity DNA Polymerase (New England Biolabs, Ipswich, MA, USA, catalog no. M0530S).

#### *Plasmids providing expression of guide RNAs under the control of the* Drosophila *U6:2 promoter*

CRISPR targets sites were identified using Target Finder (*88*) (targetfinder.flycrispr.neuro.brown.edu/). Four targets sites (two upstream of the fourth coding exon and two downstream of the seventh exon of *dG*α*o*) were selected. Complimentary oligonucleotides gRNAR2fw and gRNAR2rev were annealed and cloned into pENTR1A-DUAL-CRISPR (provided by A. Glotov, Umea University, Sweden), which was digested with Bbs I and Sap I (New England Biolabs, catalog nos. R0539S and R0569S). The resultant plasmid pDUAL-L1R2 contains two guide RNAs (gRNAs) for induction of double-strand breaks upstream of exon 4 and downstream of exon 7 of *dG*α*o*. The same approach was performed to construct pDUAL-R1L2 using the gRNAL2fw and gRNAL2rev; gRNAR1fw and gRNAR1rev oligonucleotides. The plasmids pDUAL-L1R2 and pDUAL-R1L2 combined with the donor plasmid pGao47-LattP-pBacDsRed-attPR were used for CRISPR-Cas9 step of transgenesis (fig. S11).

#### Donor plasmids for RMCE

Plasmid piB-GFP (Addgene, #13844) containing two attB sequences was used as a template for PCR amplification of the 3100-bp fragment (plasmid body flanked by attB sequences) with the primer attBcircle. *Drosophila* genomic DNA was used as a template for PCR amplification of three fragments with the primer sets attBNco_Gao47L/attBNco_Gao47R (1190 bp), attBNco_Gao47L/G203Rrev (367 bp), and G203Rfw/attBNco_Gao47R (725 bp). The amplified fragments 3100 and 1190 bp were mixed and circulated using the NEBuilder HiFi DNA Assembly Cloning Kit (p2xattB-dGaoWT). The amplified fragments 3100, 367, and 725 bp were mixed and treated identically (p2xattB-dGaoG203R). The resultant plasmid p2xattB-dGaoWT has two attB sites, which flank sequences between exons 4 and 7 of *dG*α*o* with about 100 bp of adjacent noncoding regions. p2xattB-dGaoG203R has the identical structure but bears G203R mutation in exon 5. Both plasmids were used as donor plasmids for RMCE step of transgenesis (fig. S11).

### *Drosophila* lines and germline transformation

Flies were maintained at 25°C on the standard medium. For dietary experiment, the food was supplemented with 200 μM ZnCl_2_ or 10 μM ZPT. The line y[1], sc[*], v[1], sev[21]; P{y[+t7.7] v[+t1.8]=nos-Cas9.R}attP2 expressing Cas9 in the germline under the control of the nos promoter [Bloomington *Drosophila* Stock Center (BDSC), Bloomington USA, stock #78782] was used for germline transformation in the CRISPR-Cas9 step of transgenesis (fig. S11). Transgenic flies were selected by red fluorescence in eyes provided by the expression of DsRed under the eye-specific 3xP3 promoter. The resultant fly stock *dG*α*o[47dsRed]* was combined with *P{y[+t7.7]=nos-phiC31\int.NLS}X* (BDSC, stock #34770) and balanced over *CyO*. This stock was used for the RMCE step of transgenesis (fig. S11). Transgenic flies in this case were selected by the absence of red fluorescence in eyes. The resultant alleles and *dG*α*o[WT-control]* were balanced over *CyO*. The stock *dG*α*o[G203R]/CyO* is maintained in the heterozygous state as the G203R mutation leads lethality in homozygous. The stock *dG*α*o[WT-control]* is viable and fertile in the homozygous state. This allele having the identical background to that of the mutant one was used as the wild-type control in the negative geotaxis assay and for calculation of the longevity. Germline transformation was performed as described previously (*40*). A fluorescence stereomicroscope [Zeiss SteREO Discovery.V8, Carl Zeiss, Jena Germany with the Filter Set 43 HE for the DsRed fluorescent dye detection (excitation BP 550/25; emission BP 605/70)] was used for selection of transgenic flies with/without fluorescence in the eyes.

### Molecular analysis of established fly stocks

Genomic DNA was isolated from individual flies of different genotypes as described previously (*89*). The established transgenic fly strains were verified by PCR with the primers annealing to the neighboring genomic sequences outside the homologous arms and inside the donor plasmids. PCR analysis was carried out with different primer sets: dGao47Test3 (1)/dGao47Rnew1RevLong (2), dGao47Test3 (1)/pBacWTlong (3) and pBac_rev (4)/dGao47Rnew1RevLong (2) for *dG*α*o[47dsRed];* dGao47Test3 (1)/dGao47Rnew1RevLong (2) and dGao47Test3 (1)/G203Rrev (5) for *dG*α*o[WT-control]* and *dG*α*o[G203R]/CyO* (fig. S11 and table S1). All PCRs were performed using Phusion High-Fidelity DNA Polymerase (New England Biolabs) following the manufacturer’s instructions. Intactness of inserted sequences via RMCE (stocks *dG*α*o[WT-control]* and *dG*α*o[G203R]/CyO*) was verified by sequencing. Proper expression of these alleles was verified by sequencing their cDNA performed as described previously (*40*).

### Locomotion, life span, and neurodegeneration in *Drosophila*

The negative geotaxis assay was performed as described previously (*40*) using 5- to 6-day-old flies. Further quantitative locomotion analyses were performed as described (*55*) with 15- and 30-day-old flies. Measurement of the life span was performed as described (*90*); A total of 110 males and 140 females of each genotype and on each supplemented food were monitored. Adult brain degeneration was assessed following (*47*) using costaining with Alexa Fluor 488 phalloidin (1:100; Thermo Fisher Scientific) and DAPI (1:1000) after fixation in 4% paraformaldehyde/0.5% Triton X-100 in PBS. Fluorescent images (*Z*-stacks) were acquired with a Zeiss LSM 800 Airyscan confocal microscope.

## Notes

View/request a protocol for this paper from *Bio-protocol*.
