# Supplementary material for: Restoration of the GTPase activity and cellular interactions of Gαo mutants by Zn2+ in GNAO1 encephalopathy models
Source: Sci Adv. 2022 Oct 7;8(40):eabn9350. doi: 10.1126/sciadv.abn9350 (PMC9544338; doi:10.1126/sciadv.abn9350)
Supplement: Supplementary file 1 — Figs. S1 to S13 Table S1 [file sciadv.abn9350_sm.pdf]

Science Advances
AAAS

Supplementary Materials for

Restoration of the GTPase activity and cellular interactions of Gα_o mutants by Zn^{2+} in *GNAO1* encephalopathy models

Yonika A. Larasati *et al.*

Corresponding author: Vladimir L. Katanaev, vladimir.katanaev@unige.ch

Sci. Adv. **8**, eabn9350 (2022)
DOI: 10.1126/sciadv.abn9350

The PDF file includes:

Figs. S1 to S13
Table S1
Legends for movies S1 to S3

Other Supplementary Material for this manuscript includes the following:

Movies S1 to S3

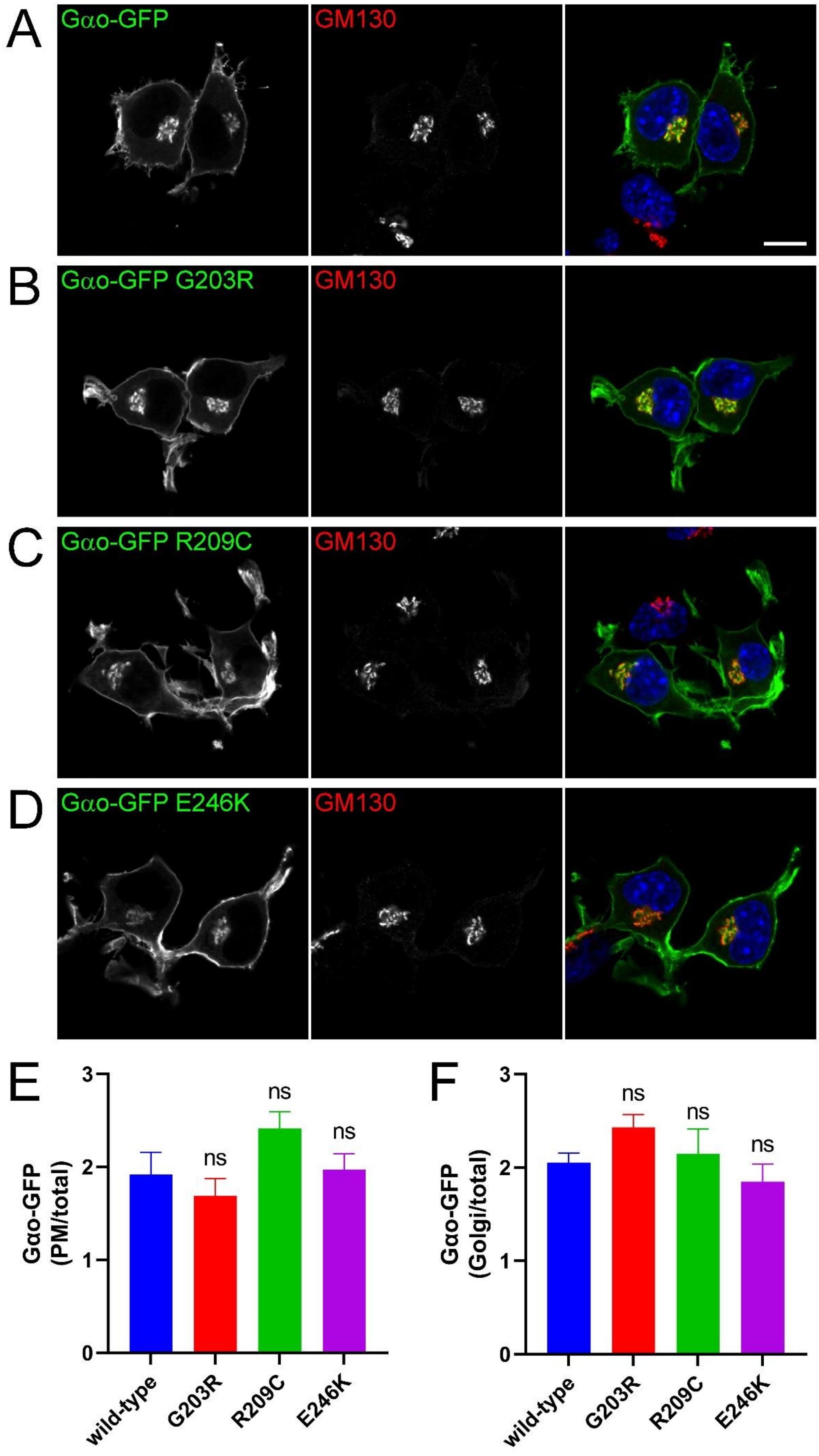
A
Gαo-GFP
GM130
B
Gαo-GFP G203R
GM130
C
Gαo-GFP R209C
GM130
D
Gαo-GFP E246K
GM130
E
Gαo-GFP (PM/total)
0
1
2
3
ns
ns
ns
wild-type
G203R
R209C
E246K
F
Gαo-GFP (Golgi/total)
0
1
2
3
ns
ns
ns
wild-type
G203R
R209C
E246K

Supplementary Figure S1. Normal intracellular localization of the [G203R], [R209C], and [E246K], mutants.

(A-D) Representative images of N2a cells expressing Gαo-GFP (C-terminally tagged) wild type (A) or the mutants G203R (B), R209C (C), and E246K (D). Gαo shows its dual localization at the plasma membrane and Golgi apparatus, and no obvious defects were observed for the Gαo encephalopathy mutants. Immunostaining against GM130 was used to label the Golgi. Scale bar, 10μm. **(E, F)** Mean fluorescence intensity ratios of GFP-fusions at the plasma membrane (PM, E) and the Golgi (F) versus total cell. Data is presented as mean ± SEM, n=9-10. ns – not significant by one-way ANOVA followed by Dunnett's multiple-comparison test.

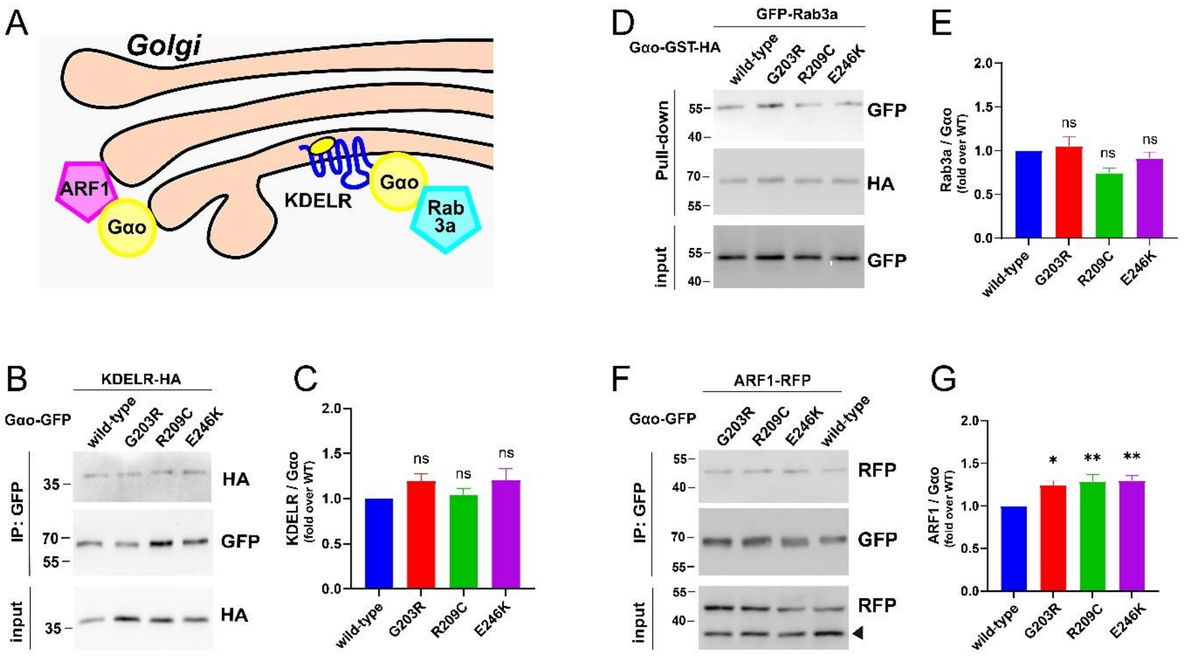

Supplementary Figure S2. Near-normal interactions of the [G203R], [R209C], and [E246K] Gαo mutants with Golgi partners.

(A) Schematic illustration of the Golgi partners of Gαo tested: KDELR, ARF1 and Rab3a. Illustration modified from ref. *(16)*. **(B, C)** N2a cells were co-transfected with HA-tagged KDELR and Gαo-GFP (C-terminally tagged) wild-type, G203R, R209C, or E246K. Immunoprecipitation (IP) of Gαo was done using a nanobody against GFP, and the co-precipitation of KDELR was analyzed by SDS-PAGE and Western Blot (B). Ab against GFP was used for detection of Gαo and against HA – for KDELR. Quantification of the co-IP of KDELR by the different Gαo constructs (C). **(D, E)** N2a cells were co-transfected with GFP-Rab3a and Gαo-GST-HA (C-terminally tagged) wild-type, G203R, R209C, or E246K mutants. Pulldown of Gαo was done using glutathione beads and samples were analyzed by SDS-PAGE and Western Blot (D). Ab against HA was used for detection of Gαo and against GFP for Rab3a. Quantification of the co-precipitation of GFP-Rab3a by Gαo-GST-HA wild type, G203R, R209C, or E246K mutants (E). **(F, G)** N2a cells were co-transfected with ARF1-mRFP and Gαo-GFP (C-terminally tagged) wild-type, G203R, R209C, or E246K mutants. Immunoprecipitation (IP) of Gαo was done using a nanobody against GFP, and samples were analyzed by SDS-PAGE and Western Blot (F). Ab against GFP was used for detection of Gαo and against RFP for KDELR. Arrowhead in 'input' indicates a degradation product of ARF1-mRFP, which did not co-precipitate with Gαo. Quantification of the co-IP of ARF1-mRFP by Gαo-GFP wild type, G203R, R209C, or E246K mutants (G). Data in (C, E, G) are presented as mean ± SEM, n=4. *p<0.05, **p<0.01, ns = not significant by one-way ANOVA followed by Dunnett's multiple-comparison test.

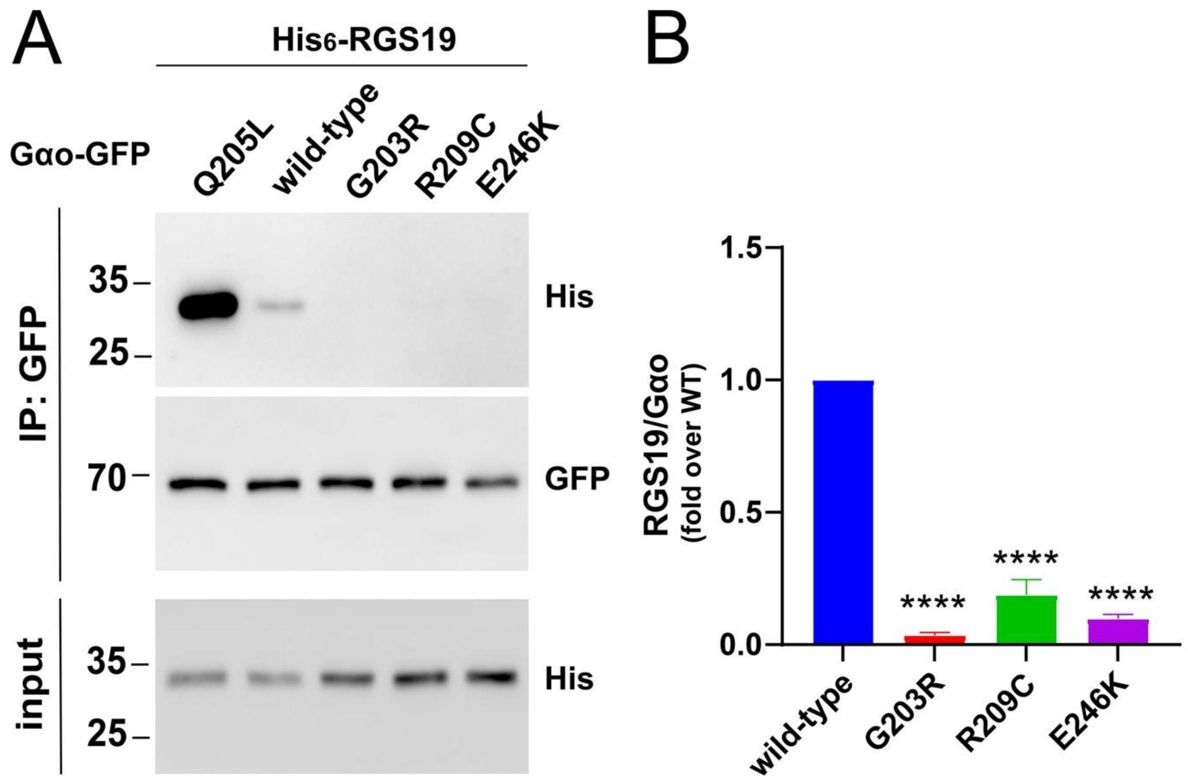

Supplementary Figure S3. Aberrant interactions of the [G203R], [R209C], and [E246K] Gαo mutants with RGS19.

N2a cells were co-transfected with His6-RGS19 and Gαo-GFP (C-terminally tagged) wild-type, G203R, R209C, E246K, or the activated Q205L mutant as control. Immunoprecipitation of Gαo was done using a nanobody against GFP, and the co-precipitation of RGS19 was analyzed by SDS-PAGE and Western Blot. Ab against GFP was used for detection of Gαo and against His_6 – for RGS19. Quantification of the co-IP of RGS19 by the different Gαo constructs. Data are mean of ≥4 biological replicates ± SEM. ****p<0.0001 by one-way ANOVA followed by Dunnett's multiple-comparison test.

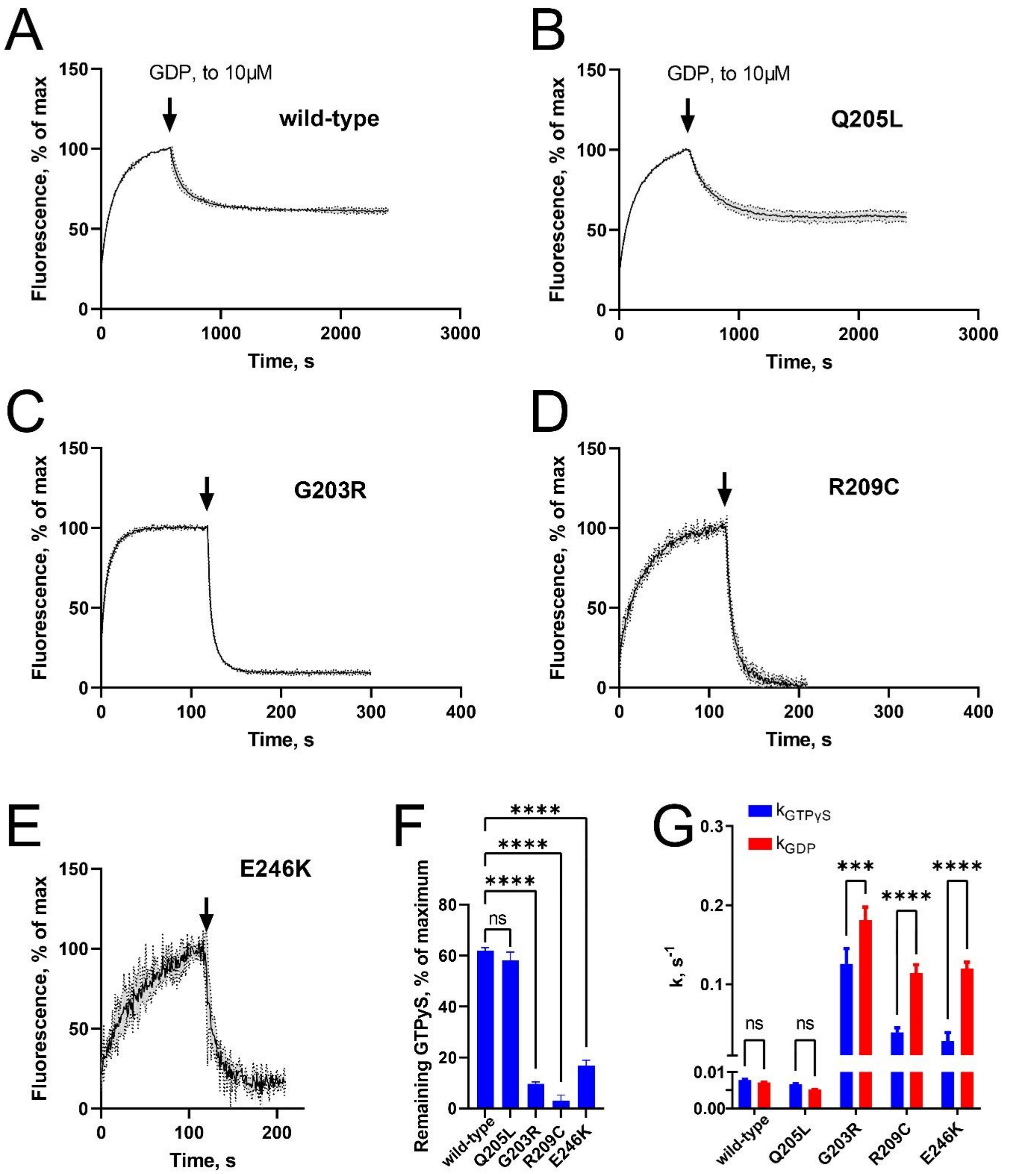

Supplementary Figure S4. The [G203R], [R209C], and [E246K] mutants are defective in holding GTP after binding.

(A-E) Representative graphs of wild-type and mutant Gαo loading by BODIPY-GTPγS followed by GDP injection to 10μM (indicated by arrowhead), which results in displacement of the fluorescence ligand and drop in fluorescence. As compared to Gαo wild-type (A) and the constitutively activated Gαo[Q205L] (B), the pathologic mutants G203R (C), R209C (D), and E246K (E) demonstrate a profound decrease in their ability to hold GTP. **(F)** Quantification of the plateau values demonstrates near complete displacement of BODIPY-GTPγS by GDP for the three pathologic mutants as compared to only ~40% loss for the wild-type protein and the Q205L mutant. (G) Quantification of apparent rate

constants of association with BODIPY-GTPγS ($k_{GTPγS}$, identical to k_{bind} in Fig. 1C) and its loss upon outcompeting by GDP (k_{GDP}). While the two kinetic constants are equal for Gαo wild-type and Gαo[Q205L], k_{GDP} is significantly higher than $k_{GTPγS}$ for the three pathologic mutants. The data are presented as an average from N=2-3 independent experiments, SEM are indicated as filled area around the curve or error bars. Statistical assessment performed by one-way ANOVA followed by Dunnett's multiple comparison test (F) or two-way ANOVA followed by Tukey's multiple comparison test (G), the significance is shown as ***$p<0.001$, ****$p<0.0001$; ns = not significant.

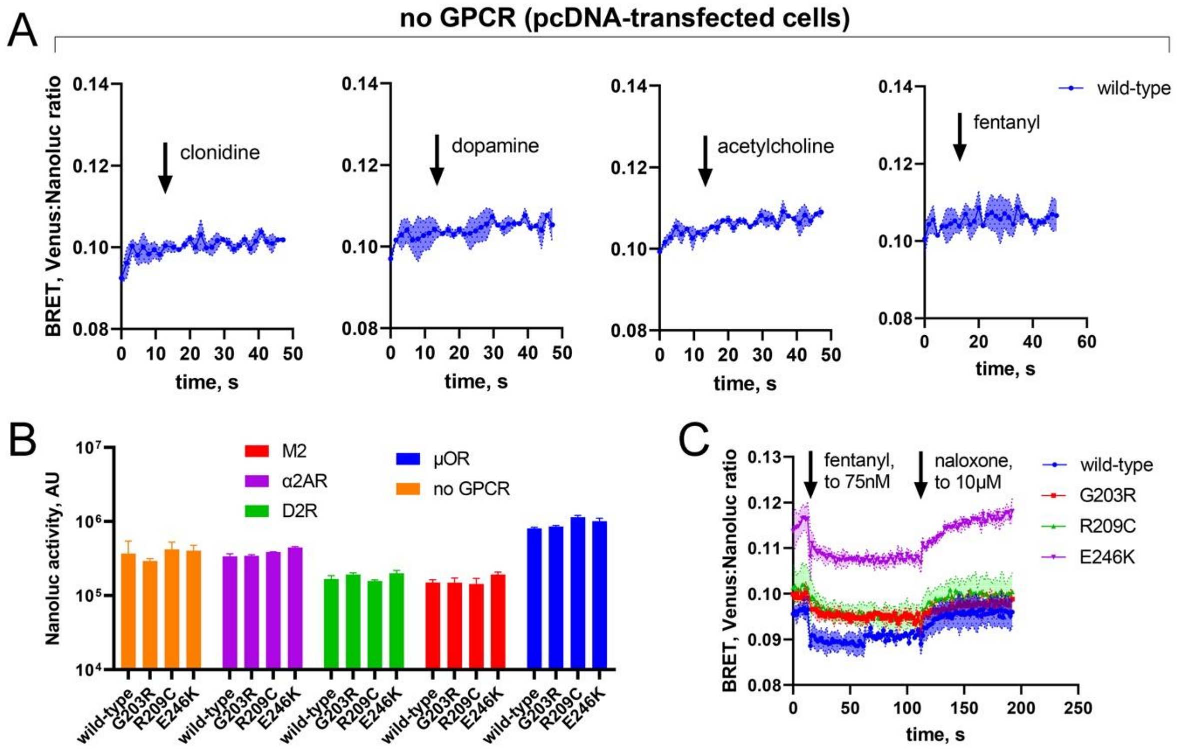

Supplementary Figure S5. Neuronal GPCR signaling controls.

(A) Injection of the indicated GPCR agonists in the HEK293T cells transfected with the construct encoding wild-type Gαo alone does not change BRET ratio, indicating absence of the basal activity of these GPCRs. **(B)** Average values of nanoLuciferase activity (average of 10 first readings prior to stimulation) demonstrate equivalence of the transfection levels among Gαo variants. **(C)** Upon injection of the μ-opiod receptor antagonist naloxone (10 μM), the BRET ratio reduced after addition of the fentanyl agonist returns to its original value indicating full capacity of Gαo wild-type as well as the mutants to recombine with Gβγ. Data presentation and agonist concentrations are as in Fig. 3.

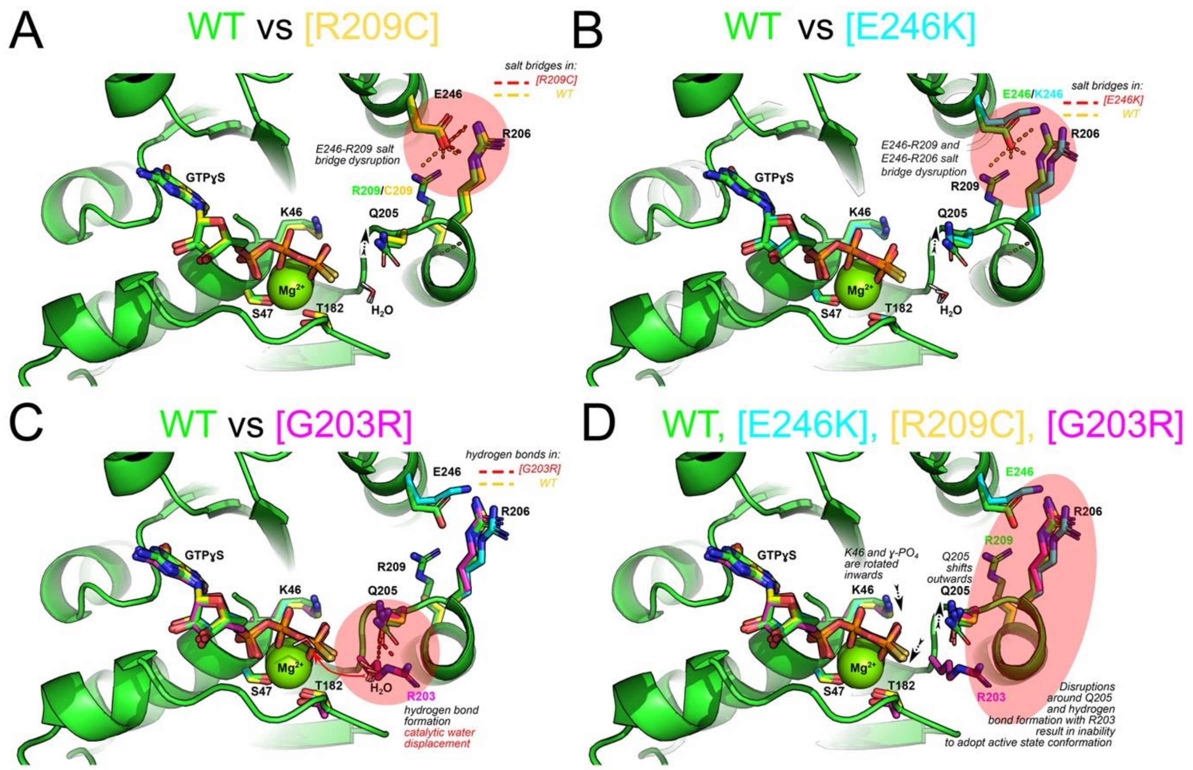

Supplementary Figure S6. Mutations [G203R], [R209C], and [E246K] in Gαo affect the immediate surroundings of Q205, the key residue for GTP hydrolysis.

The panels show the active site of wild type or mutant Gαo energy-minimized models. All the panels show the cartoon of the backbone of wild type Gαo (WT, green) overlaid with color-coded side chains (C-atoms colored only) of mutant proteins. Indicated residues are the ones which have the major role in the complex formation with Mg^{2+} (S47, T182), orientation of γ-phosphate (K46) and orientation of Q205 (R206, R209, E246). **(A, B)** Mutations R209C and E246K disrupt either one (A) or both (B) salt bridges formed in the GTP-bound Gαo between the α2 and α3 helices and required to adopt the active conformation. **(C)** Mutation G203R results in formation of a new hydrogen bond between side chains of R203 and Q205. Additionally, it results in displacement of the water molecule required for the hydrolysis due to the resulting steric hindrance (red arrow). **(D)** Similarity of the changes in the 3 mutants: in all of them, the Q205 side chain is significantly moved outwards of the plane of view, whereas the γ-phosphate and the K46 side chain are displaced inwards thus increasing the distance between Q205 and the γ-phosphate.

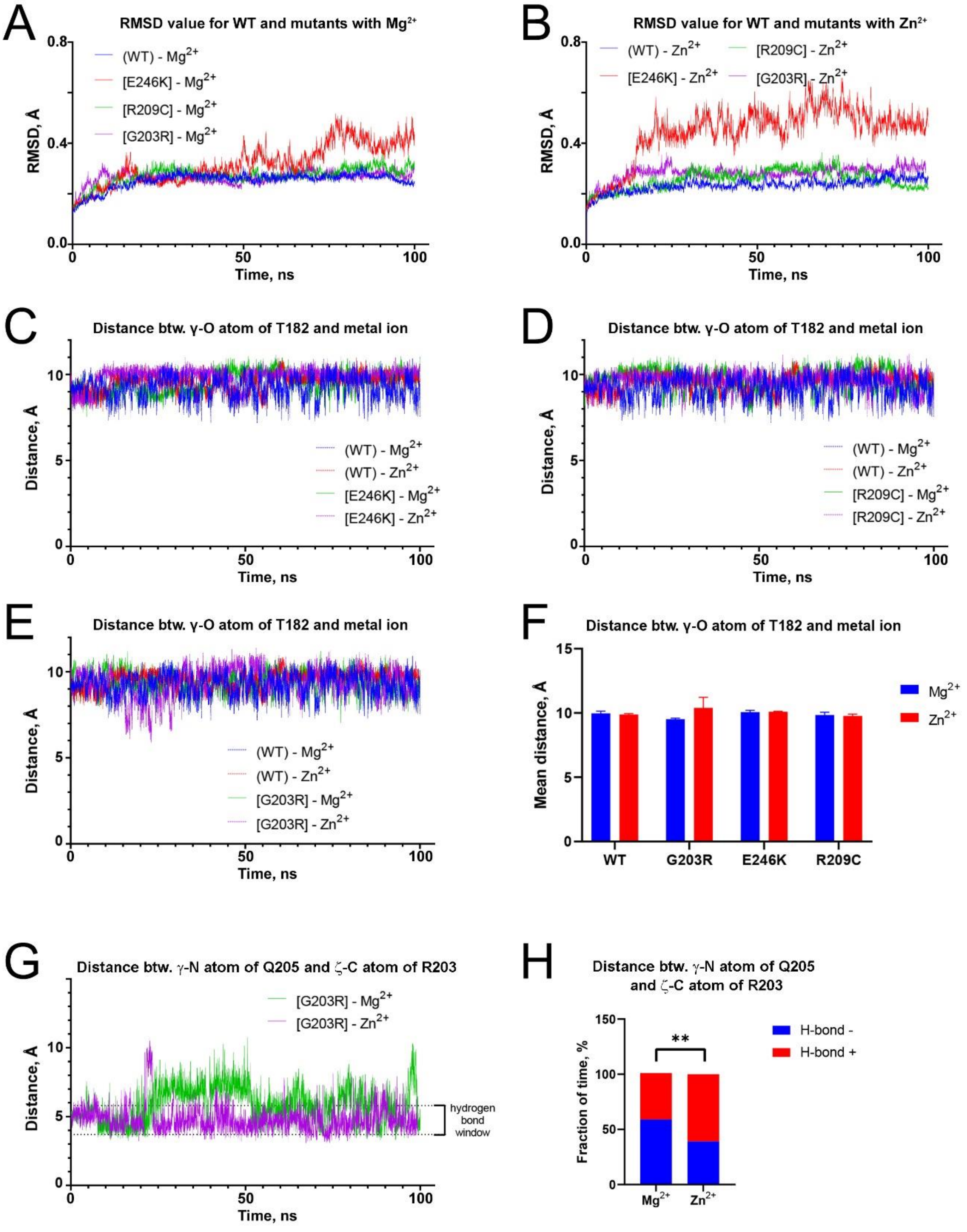

Supplementary Figure S7. Analysis of global stability, T182 residue, and R203-Q205 residues in Gαo wild type and mutants in the Mg^{2+}- and Zn^{2+}-bound GTP states by molecular dynamics.

(A, B) RMSD plots reflect overall changes of the wild type (WT) Gαo or the three mutants with Mg^{2+} (A) or Zn^{2+} (B) in the active site. Only the [E246K] mutant demonstrates significant global rearrangements as compared to the wild type (see also Supplementary Movie S1). These rearrangements are somewhat increased when Mg^{2+} is replaced by Zn^{2+} (see also Supplementary Movie S2). **(C-F)** Analysis of the distance between the γ-O atom of T182 and the metal ion center in the wild type or mutant Gαo proteins

reveals no significant influence of Zn^{2+} / Mg^{2+} substitution on the position of this residue in any Gαo form. (C) to (E) are individual MD simulations, and (F) is the summary of the last 50ns of three independent MD simulations presented as mean ± SD. **(G)** Comparison of the distance between the γ-N atom of Q205 residue and ζ-C atom (center of guanidino group) of R203 in Gαo[G203R] with Mg^{2+} or Zn^{2+} in the active site over 100ns of molecular dynamics simulation. The limits of the "hydrogen bond window" are marked on the graph. Note that these atoms are not directly involved in the hydrogen bond formation, however this distance provides the best measure of the R203-Q205 H-bonding because Q205' carbonyl oxygen forms H-bonds with hydrogen of different nitrogens of R203' guanidino group throughout simulation. The results indicate that Zn^{2+} stabilizes this bond, rather than disrupts it. **(H)** Fraction of time that enables H-bond formation between γ-N atom of Q205 and ζ-C atom of R203 in G203R mutant bound to different metal ions calculated from the last 50ns of three independent MD simulations, analyzed statistically with chi-squared test, $^{**}p<0.01$.

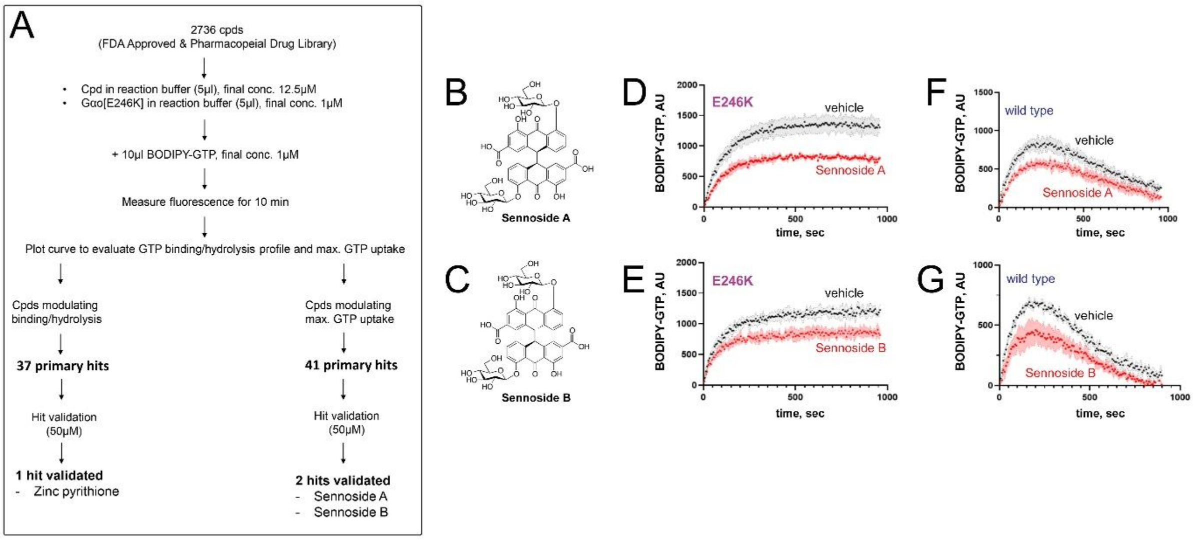

Supplementary Figure S8. High-throughput screening to recover the GTPase activity of Gαo[E246K].

(A) The scheme of high-throughput screening (HTS) performed by measuring GTP binding and hydrolysis by Gαo[E246K]. **(B, C)** In addition to ZPT, HTS and hit validation resulted in two other compounds: sennoside A and sennoside B. **(D, E)** Representative curves of BODIPY-labeled GTP binding and hydrolysis by Gαo[E246K] in the presence of 50µM sennoside A (D) or sennoside B (E). **(F, G)** Representative curves of BODIPY-labeled GTP binding and hydrolysis by wild type Gαo in the presence of 50µM sennoside A (F) or sennoside B (G). It is apparent that the sennosides affect wild type Gαo. Data in (D-G) are mean of two biological replicates ± SEM and is presented as in Fig. 2B, C.

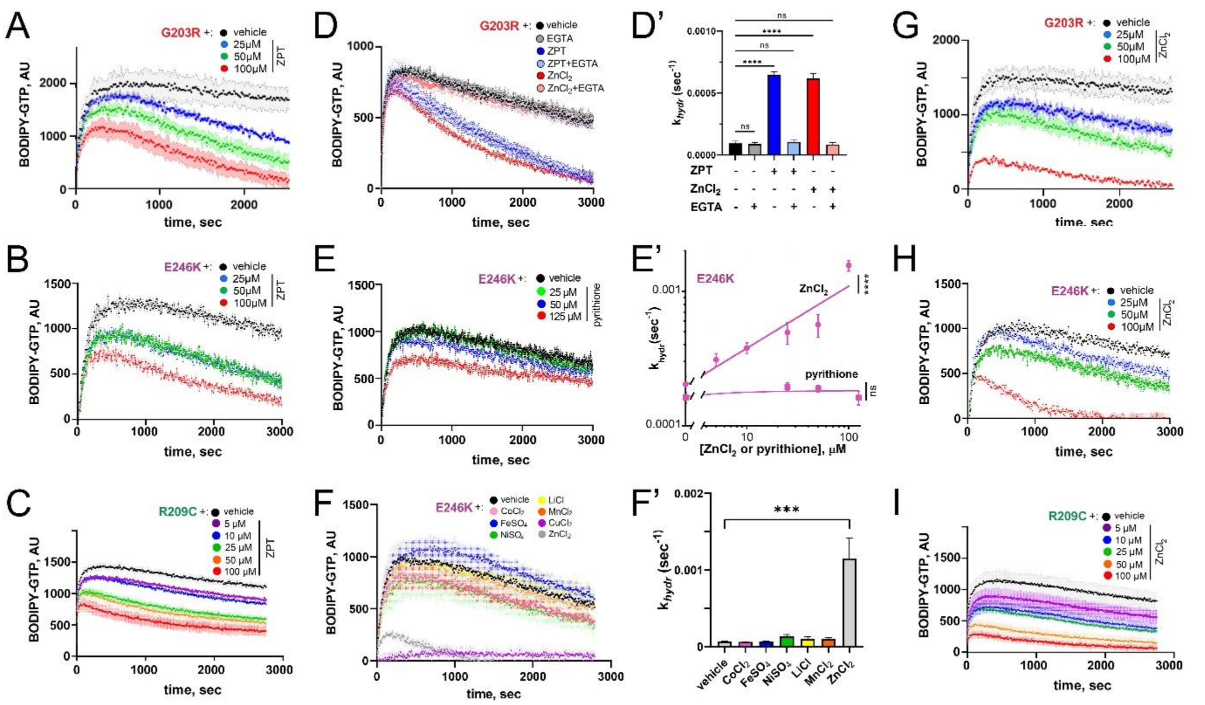

Supplementary Figure S9. ZPT and Zn^{2+} restore the GTPase activity of the three encephalopathy Gαo mutants.

(A-C) Representative curves of BODIPY-labeled GTP binding and hydrolysis by Gαo[G203R] (A), Gαo[E246K] (B), and Gαo[R209C] (C) in the presence of indicated concentrations of ZPT. **(D, D')** Restoration of the GTPase activity of Gαo[G203R] by 50μM ZPT or $ZnCl_2$ was abolished by 1mM EGTA. (D) provides representative curves of BODIPY-labeled GTP binding and hydrolysis, (D') – quantification; data are mean ± SEM of ≥3 biological replicates. ****$p<0.0001$; ns = not significant by two-way ANOVA with Sidak corrections for multiple comparisons. **(E, E')** Unlike ZPT or $ZnCl_2$, pyrithione was ineffective in restoring the GTPase activity of Gαo[E246K]. (E) provides representative curves of BODIPY-labeled GTP binding and hydrolysis, (E') – quantification as in Fig. 2E; note the log scale in the X- and Y-axes. **(F, F')** Unlike $ZnCl_2$, various salts of other metals at 100μM failed to restore the GTPase activity of Gαo[E246K]. (F) provides representative curves, and (F') – quantification (mean of two experiments ± SEM). ***$p<0.001$ by one-way ANOVA followed by Dunnett's multiple comparison test. **(G-I)** Representative curves of BODIPY-labeled GTP binding and hydrolysis by Gαo[E203R] (G), Gαo[E246K] (H), and Gαo[R209C] (I) in the presence of indicated concentrations of $ZnCl_2$.

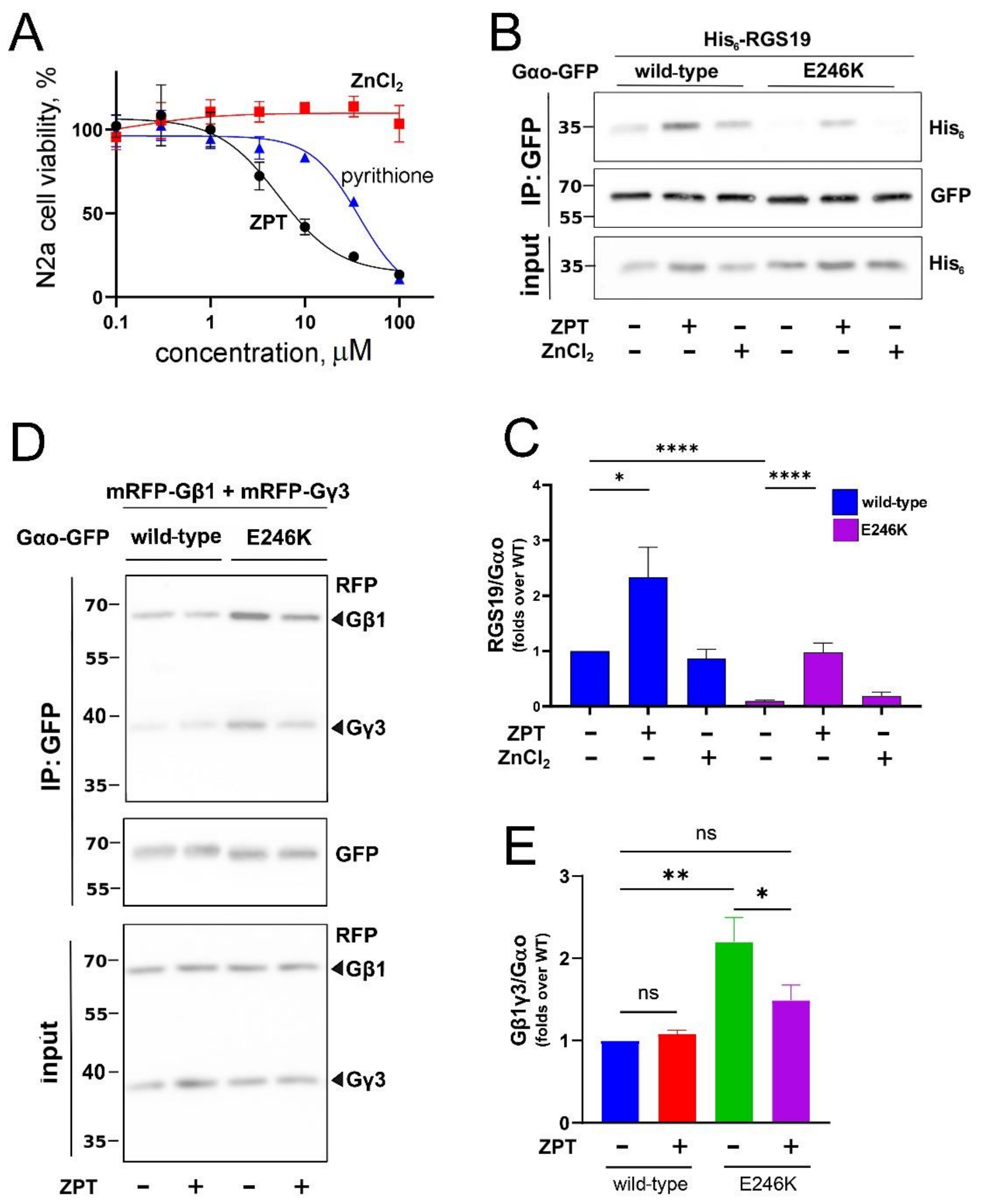

Supplementary Figure S10. ZPT normalizes cellular interactions of Gαo[E246K] with RGS19 and Gβγ.

(A) Acute toxicity of ZPT, $ZnCl_2$, and pyrithione were determined on N2a cells. N2a cells were treated with 0.1-100 µM of compounds for 3 h before being subjected to the MTT assay. **(B, C)** N2a cells were co-transfected with His_6-RGS19 and Gαo-GFP (C-terminally tagged), wild type or [E246K]. The next day, cells were treated with DMSO, 1 µM ZPT, or 100 µM $ZnCl_2$ for 3 h before being subjected for immunoprecipitation (IP). IP of Gαo was done using a nanobody against GFP, and the co-precipitation of RGS19 was analyzed by SDS-PAGE and Western Blot. Abs against GFP and His_6-tag were used for the detection of Gαo and RGS19, respectively (B). Quantification of the co-IP of His_6-RGS19 by Gαo-GFP wild type and E246K, normalized to the binding levels of wild-type Gαo (C). Data are mean of at least three biological replicates ± SEM. *$p<0.05$, ****$p<0.0001$ on log-transformed data by two-way ANOVA with Sidak corrections for multiple comparisons. **(D, E)** N2a cells were co-transfected with mRFP-Gβ1, mRFP-Gγ3, and Gαo-GFP (C-terminally tagged) wild-type and [E246K]. The next day,

cells were treated with DMSO or 1 µM ZPT for 3 h before subjected for immunoprecipitation (IP). IP of Gαo was done using a nanobody against GFP, and the co-precipitation of Gβ1γ3 was analyzed by SDS-PAGE and Western Blot. Ab against GFP was used for detection of Gαo and against mRFP (RFP) – for Gβ1γ3 (arrowheads). Quantification of the co-IP of Gβ1γ3 by the different Gαo constructs and treatment with ZPT. Data are mean of ≥3 biological replicates ± SEM. ns: not significant, *p<0.05; **p<0.01 by two-way ANOVA with Sidak corrections for multiple comparisons.

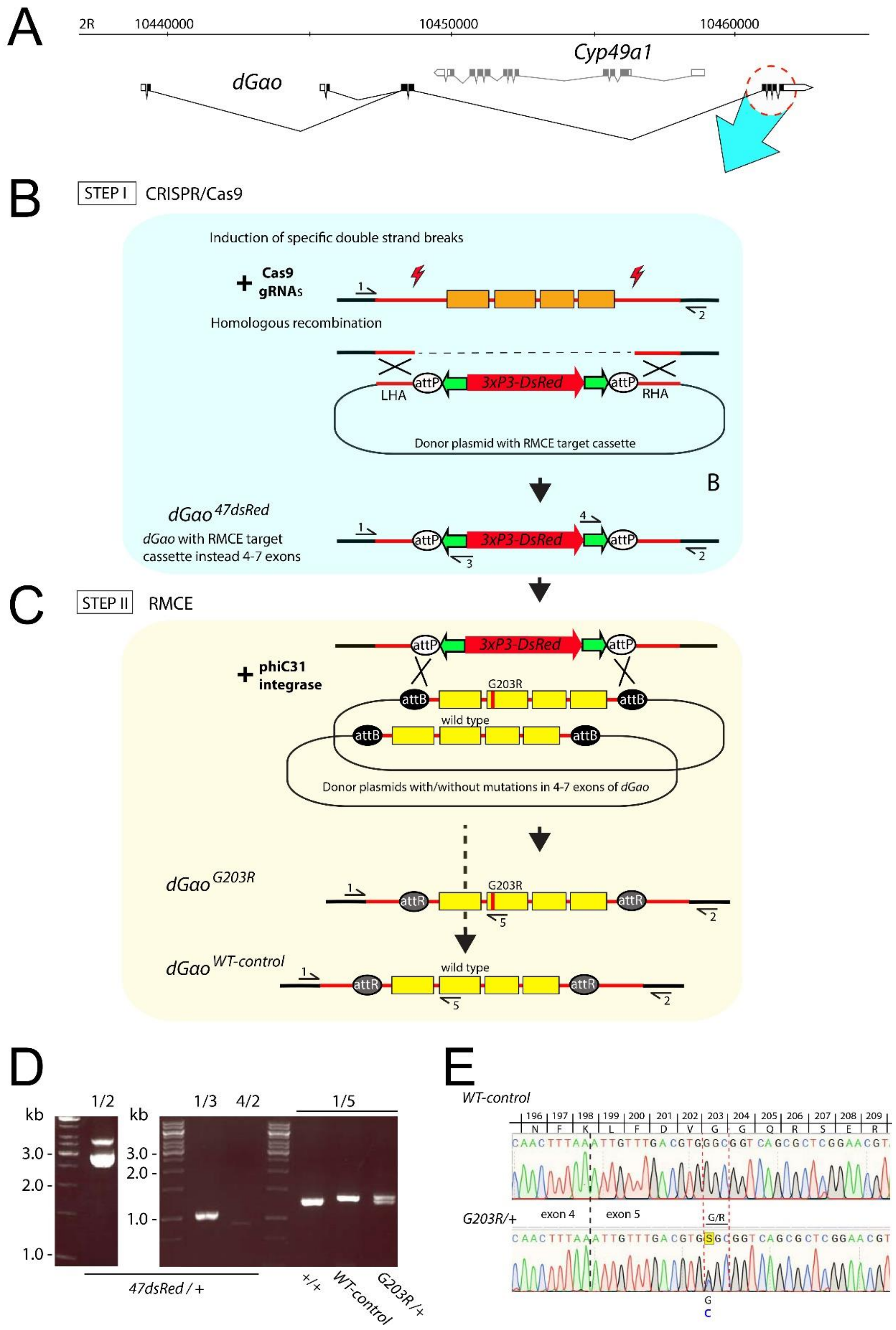

Supplementary Figure S11. Scheme of creation and verification of *dGαo* mutants.

(A) Gene map of *dGαo*, including the gene *Cyp49a1* inserted in the intronic region, on the chromosome arm 2R of *D. melanogaster*, based on the GBrowse function of Flybase. Numbers on top are the chromosome' nucleotide numbers. **(B, C)** A two-step strategy for bringing point mutations in *dGαo* was applied. The first step (blue background, B) is creation of flies with the target cassette instead of exons

4-7 of *dGαo*. The primers for validation of the correct donor sequence integration in the genome are designated with black horizontal arrows and marked with numbers (see Methods for the respective primer sequences). Left (LHA) and right homologous arms (RHA) in the donor plasmids were required for the homologous recombination with the genomic sequences during the CRISPR/Cas9 gene editing. The second step (orange background, C) is creation of mutant alleles via the recombinase-mediated cassette exchange (RMCE) using phiC31-integrase. The ovals marked as attP, attB and attR are designated recognition sequences for phiC31-integrase (attP, attB) and the result of recombination between them (attR). The vertical red line in the 5th exon designates the sequence with mismatches that lead to the G203R mutation. The sequences recognized by the primers for validation of transgenesis are shown with black horizontal arrows and are numbered. **(D)** Agarose gels with PCR products obtained with primers numbered above each lane (see Supplementary Table S1 for sequences). Two bands on the left panel correspond to two alleles: *wt* (2755 bp) and *47dsRed* (3630 bp). The difference in length of PCR products obtained with primers 1/5 (right panel) is the result of the presence of attR site (80 bp) in transgenic flies but not in *wt* (+/+) flies. **(E)** Sequences of RT-PCR products for the *G203R/+* mutant and the *WT-control* alleles. Codons, amino acids with their numbers are shown above the sequence of the *WT-control* allele. Black dashed vertical line demarcates the 4th and 5th exons. Red dashed vertical lines designate the codon for the 203rd amino acids of Gαo.

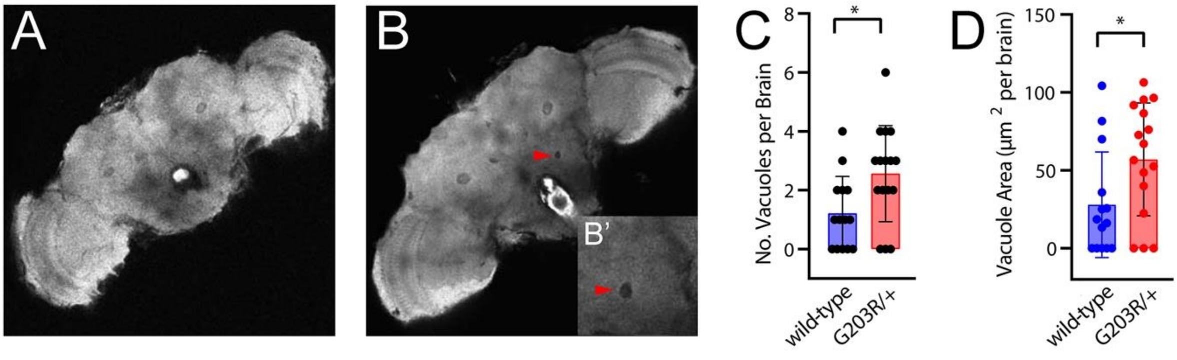

Supplementary Figure S12. Limited brain degeneration in *[G203R]/+* flies.

(A, B) Integrity of brains of 35 days-old adult flies, wild-type (A) and *[G203R]/+* (B), was analyzed by confocal microscopy after immunostaining with DAPI and Alexa Fluor 488 phalloidin. Representative cross-sections are shown as 200x200 µm images. In addition to vacuoles stereotypically observed in adult brains, additional vacuoles signifying areas of degeneration could be observed (arrowheads in (B, B'); insert (B') is 80x80 µm). **(C, D)** Quantification of 14 wild-type and 16 *[G203R]/+* brains (1:1 male/female) shows that both the number of degenerative vacuoles (C) and the total degeneration area (D) are increased in the mutant condition. Data are mean ± SD. *$p<0.05$ by the *t*-test.

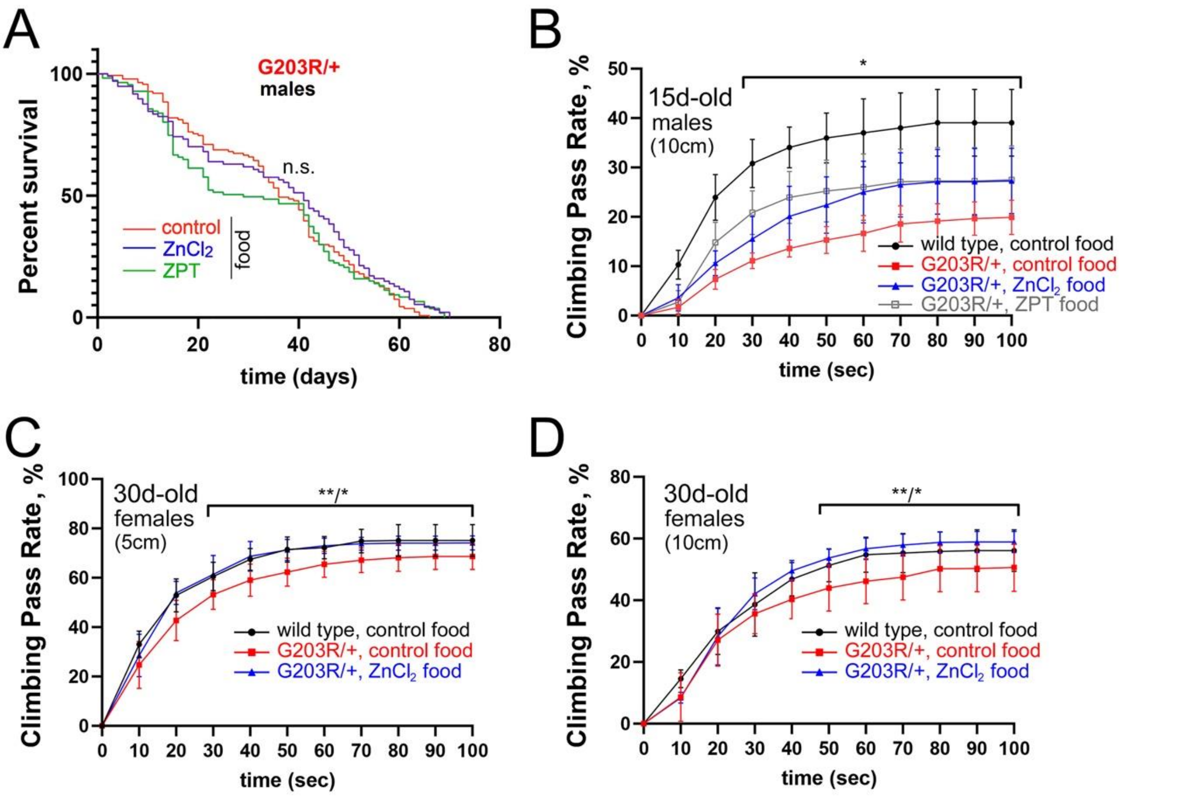

Supplementary Figure S13. Effects of dietary Zn^{2+} on the lifespan and locomotion of *G203R/+ Drosophila*.

(A) The lifespan of male *G203R/+* flies is not influenced by dietary supplementation of $ZnCl_2$ or ZPT. Data presentation and analysis as in Fig. 4C, F. Median survivals are calculated as 29days (ZPT-supplemented food), 36 days (control food), and 41 days ($ZnCl_2$-supplemented food); Mantel-Cox and Gehan-Breslow-Wilcoxon tests both show no statistical significance among the three conditions ('n.s.'). **(B)** Wild-type and *G203R/+* male flies, 15 days-old grown on control, $ZnCl_2$- or ZPT-supplemented food, were tested in a video-recorded quantitative negative geotaxis assay (see Supplementary Movie S3) with 10-sec measurements of the number of flies climbing above 10cm over the entire period of 100 seconds. **(C, D)** Old (30 days) female flies, wild-type and *G203R/+*, raised on control or $ZnCl_2$-supplemented food, were tested in the quantitative negative geotaxis assay, monitoring flies climbing above 5cm (C) and 10cm (D). As opposed to the measurements of climbing above 18cm (Fig. 4H), significant rescuing effect of the $ZnCl_2$ supplementation could be observed. Data in (B-D) are shown as

mean ± SD, n = 7-10. Stars indicates the t-test' p-value < 0.01 (*) and < 0.001 (**) for the indicated time points for the differences between control food and $ZnCl_2$ supplementation for the mutant flies.

Supplementary Table S1. Oligonucleotides used in the molecular analysis and cloning. Numbers in brackets after the primers' names represent how they are designated in Supplementary Fig. S11.

Ep203fw	GACGTCAGAGGCCAGCGATCTGA
Ep203rev	TCAGATCGCTGGCCTCTGACGTC
Ep209fw	AGCGATCTGAATGCAAGAAGTGGATCCA
Ep209rev	TGGATCCACTTCTTGCATTCAGATCGCT
Ep246fw	ACCGCATGCACAAGTCTCTCAT
Ep246rev	ATGAGAGACTTGTGCATGCGGT
attPfwRI	TAGTGTCTTCGGGGCCGAATTCAGAAGCGGTTTTCGGGAGTAGT
attPrevHpaI	TATCTTTCTAGGGTTAACATGTCGGCGACCCTAC
attPfwKpnI	TGGTCTTCTTTTCCCGGTACCAGAAGCGGTTTTCGGGAGTAGT
dGao47Lrev	CGACAT*GAATTC*TGCCAAGACTTGAGCAAACAAT
dGao47Lfw	ACCGCA*GAATTC*GAGCATGACTTAGCCAATTTCC
dGao47Rfw	ACACAA*GGTACC*GGTATTAAAGAGCAGCGCGGG
dGao47Rrev	ACAACTGTATGCAATGTTGGCGCTTGTG
attBcircle	CCATGGGTGAGGTGGAGTAC
attBNco_Gao47L	TACTCCACCTCACCCATGGGCGGAAAATTGAGAATGGACGGGT
attBNco_Gao47R	TACTCCACCTCACCCATGGTTACGGTGTTCCCTGCTAACGCCC
G203Rfw	TTTGACGTGCGCGGTCAGCGCT
G203Rrev (5)	AGCGCTGACCGCGCACGTCAAA
dGao47Rnew1RevLong (2)	GACCACACGCTGACACTCAATCAAATGAATA
dGao47Test3 (1)	CGAATATCGGGCATTAGCTTTGA
pBacWTlong (3)	CCGATAAAACACATGCGTCAATTT
pBac_rev (4)	GAGAGAGCAATATTTCAAGAATGC
loxPfw	GGCCATAACTTCGTATAATGTATGCTATACGAAGTTAT
loxPrev	GGCCATAACTTCGTATAGCATACATTATACGAAGTTAT
gRNAR2fw	CTTCGCAGAATGGGGGCGTTAGCA
gRNAR2fw	AAACTGCTAACGCCCCCATTCTGC
gRNAL1fw	TTCGCTCAAGTCTTGGCAGATAC
gRNAL1rev	AACGTATCTGCCAAGACTTGAGC
gRNAL2fw	CTTCGAATGGACGGGTGGAAGGGT
gRNAL2rev	AAACACCCTTCCACCCGTCCATTC;
gRNAR1fw	TTCGCTGCTCTTTAATACCGTTA
gRNAR1rev	AACTAACGGTATTAAAGAGCAGC
3xHAfw	CCGGTCGCCACCATGTACCCATACGATGTTCCTGACTATGCGGG CTATCCCTATGACGTCCCGGACTATGCAGGATCCTATCCATATGAC GTTCCAGATTACGCTAGTTGAAAGC
3xHArev	GGCCGCTTTCAACTAGCGTAATCTGGAACGTCATATGGATAGGAT CCTGCATAGTCCGGGACGTCATAGGGATAGCCCGCATAGTCAGG AACATCGTATGGGTACATGGTGGCGA

Supplementary Movie S1. Overlay of the overall protein structure over 100ns of molecular dynamics simulations in the three mutants and wild type Gαo protein. Note a significant displacement of the all-helical domain in the [E246K] mutant and lack of such rearrangements in the other Gαo forms.

Supplementary Movie S2. Overlay of the overall protein structure over 100ns of molecular dynamics simulation of wild type or the three Gαo mutant proteins loaded with Mg^{2+} or Zn^{2+}. Note aggravation of the all-helical domain displacement in the [E246K] mutant and lack of thereof in the other Gαo forms.

Supplementary Movie S3. Video-recorded quantitative negative geotaxis assay, with the example of 15 days-old female *G203R/+* flies raised on the control (left cylinder) *vs.* the $ZnCl_2$-supllemented (right cylinder) food. Semi-automated quantification of the number of flies passing 5cm (blue line), 10cm (red line) and 18cm (black line) from the bottom every 10sec for the overall period of 100sec (the first 30sec are shown on the video) is performed. Zinc supplementation strongly increases the locomotion of the mutant flies.
